# Systematic review and meta-analysis of mortality risk prediction models in adult cardiac surgery

**DOI:** 10.1093/icvts/ivab151

**Published:** 2021-05-26

**Authors:** Shubhra Sinha, Arnaldo Dimagli, Lauren Dixon, Mario Gaudino, Massimo Caputo, Hunaid A Vohra, Gianni Angelini, Umberto Benedetto

**Affiliations:** 1 Bristol Heart Institute, Translational Health Sciences, University of Bristol, Bristol, UK; 2 Weill Cornell Medical College, Cornell University, New York, USA

**Keywords:** Mortality, Cardiac surgery, Prediction, European System for Cardiac Operative Risk Evaluation, Society of Thoracic Surgeons

## Abstract

**OBJECTIVES:**

The most used mortality risk prediction models in cardiac surgery are the European System for Cardiac Operative Risk Evaluation (ES) and Society of Thoracic Surgeons (STS) score. There is no agreement on which score should be considered more accurate nor which score should be utilized in each population subgroup. We sought to provide a thorough quantitative assessment of these 2 models.

**METHODS:**

We performed a systematic literature review and captured information on discrimination, as quantified by the area under the receiver operator curve (AUC), and calibration, as quantified by the ratio of observed-to-expected mortality (O:E). We performed random effects meta-analysis of the performance of the individual models as well as pairwise comparisons and subgroup analysis by procedure type, time and continent.

**RESULTS:**

The ES2 {AUC 0.783 [95% confidence interval (CI) 0.765–0.800]; O:E 1.102 (95% CI 0.943–1.289)} and STS [AUC 0.757 (95% CI 0.727–0.785); O:E 1.111 (95% CI 0.853–1.447)] showed good overall discrimination and calibration. There was no significant difference in the discrimination of the 2 models (difference in AUC −0.016; 95% CI −0.034 to −0.002; *P* = 0.09). However, the calibration of ES2 showed significant geographical variations (*P* < 0.001) and a trend towards miscalibration with time (*P*=0.057). This was not seen with STS.

**CONCLUSIONS:**

ES2 and STS are reliable predictors of short-term mortality following adult cardiac surgery in the populations from which they were derived. STS may have broader applications when comparing outcomes across continents as compared to ES2.

**REGISTRATION:**

Prospero (https://www.crd.york.ac.uk/PROSPERO/) CRD42020220983.

**Figure:**
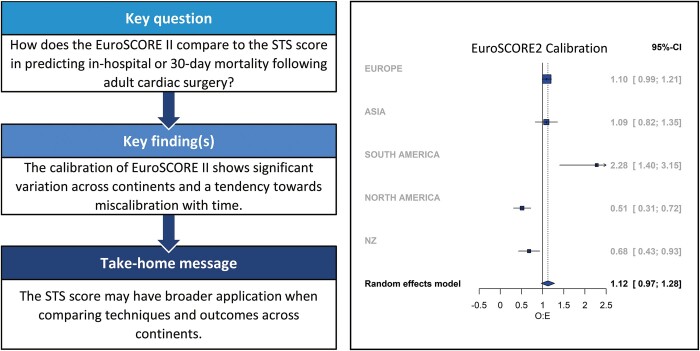

## INTRODUCTION

Cardiac surgery carries an inherent risk of perioperative mortality and morbidity. This varies considerably depending on the patients’ characteristics, baseline pathology and planned surgical intervention. Prediction models have been created [1–6] to quantify this risk. These models are utilized when counselling patients, discussing patients within the multi-disciplinary team, for benchmarking performance and more recently in guidelines for the management of aortic stenosis and deciding between surgical or transcatheter treatments [7, 8]. Present models predominantly quantify the risk of death in the short term. The most cited models are the European System for Cardiac Operative Risk Evaluation (ES) [1, 2, 9] and the Society of Thoracic Surgeons (STS) score [10, 11].

There is no guidance at present on which is the optimum score to utilize in a given clinical or research setting and concerns have arisen regarding the degree of applicability of a specific model to a localized population given the heterogenous populations from which they were originally derived. This leaves clinicians with the difficult decision of choosing which model to utilize when reporting and comparing outcomes. The relative performance of these models is thus the focus of this systematic review. We aim to build on previous work by using dedicated statistical methods to evaluate the comparative discrimination and calibration of the ES2 and STS not only in the wider cardiac surgery spectrum but also as they are applied to specific subgroups of the population. We believe that this is the most thorough comparison of these models.

## METHODS

The data and scripts that support the findings of this study are available from the corresponding author upon reasonable request.

### Systematic review

We report on the original papers and subsequent external validations available and draw comparisons between the models’ discriminatory power, as defined by the area under the receiver operator curve (AUC) or *C*-statistic, and their calibration, as defined by the ratio of the observed-to-expected mortality (O:E) within 30 days of the operation or the same hospital admission. Longer-term follow-up data were not included in the analysis to allow parity among studies and with the originally published papers on STS and ES2. A systematic literature review and meta-analysis of the above findings followed the Preferred Reporting Items for Systematic Reviews and Meta-Analyses [12] and Meta-analysis Of Observational Studies in Epidemiology principles [13].

**Figure 1:**
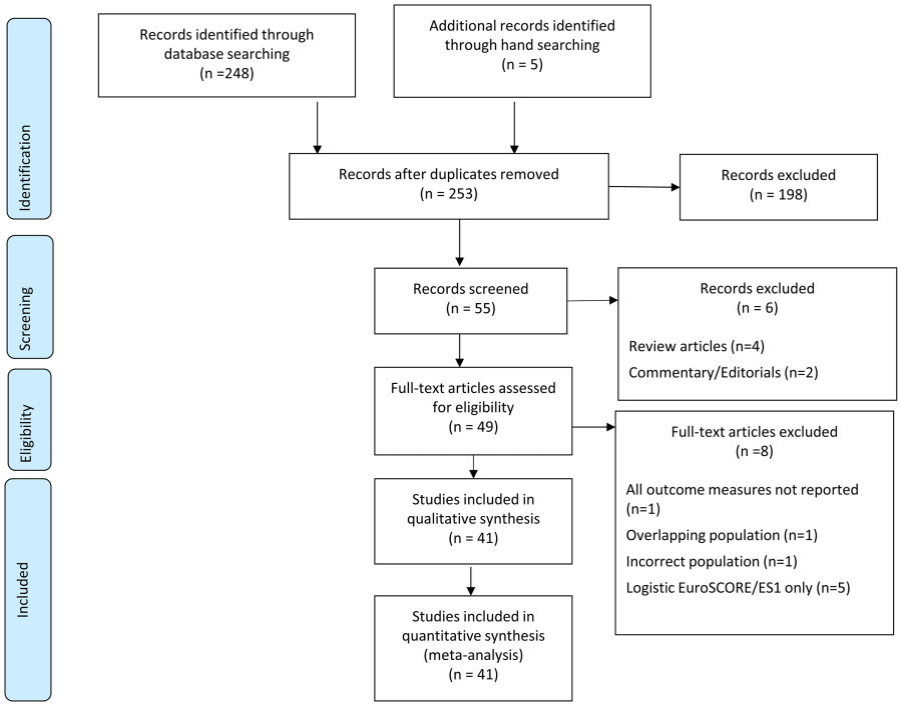
Preferred Reporting Items for Systematic Reviews and Meta-Analyses flowchart.

Our librarian conducted a literature search, restricting articles to those translatable into English and referencing adults only, using the described search string (Supplementary Material, Table S1). We also hand-searched the reference lists of papers identified but did not contact the authors. Excluded papers and rationale for exclusion have been noted (Fig. 1 and Supplementary Material, Table S2). If studies performed subgroup analysis such that the AUC or predicted mortality was not available for the whole dataset, then the subgroups were treated as independent populations. Institutes reporting on multiple occasions but utilizing different populations of patients were also treated as independent populations. The search is updated to 29 October 2020. Papers were screened and data extracted independently by 3 reviewers (SS/AD/LD). Outliers and studies with a high risk of bias were included the primary analysis following discussion between 2 authors (SS/UB). SS/UB had full access to all the data in the study and take responsibility for its integrity and the data analysis. The data extraction items were based on the CHARMS checklist [14] and the risk of bias was assessed using the PROBAST tool [15, 16] (Prospero ID: CRD42020220983).

Databases searched: MEDLINE (1946 to present), CINAHL (1981 to present), Embase (1974 to present) and EmCare (1946 to present).

Preferred Reporting Items for Systematic Reviews and Meta-Analyses diagram: Fig. 1.

Risk of bias assessment: Supplementary Material, Table S3.

Low risk of bias: 17 papers.

Uncertain risk of bias: 2 papers.

High risk of bias: 24 papers.

### Statistical analysis

Data were extracted as frequency and percentage for categorical variables and mean and standard deviation for continuous variables. The outcomes were AUC and O:E. Two separate analyses were conducted. First, we reviewed each score in turn and provided pooled estimates of AUC and O:E for comparison in accordance with previously published guidance [16–18]. It was assumed that variation in these parameters across studies was prone to between-study heterogeneity, due to the varied case-mix of populations studied, and thus, a random effects model was utilized [17]. The standard error of the AUC was calculated using Newcombe Method 4 [19]:

$$\hat{\text{V}}\text{ar}(\hat{c})=\frac{\hat{c}(1-\hat{c})\left[1+n*\frac{1-\hat{c}}{2-\hat{c}}+\frac{m*\hat{c}}{1+\hat{c}}\right]}{mn}$$

*ĉ* is the estimated AUC, *n* is the number of observed events and *m* is the number of non-events, m* = n* = [1/2 (*m* + *n*)] − 1).

Analysis was conducted using R (version 4.0.3). Meta-analysis models were formed using R-package ‘metamisc’ [17] and ‘metafor’ [20] and results displayed as forest plots. We reported 95% prediction interval (PI), which takes into account the between-study heterogeneity [17].

Second, for studies reporting ES2 and STS, we established pooled estimates of discrimination (AUC) and calibration (O:E) for each model and compared the confidence intervals (CIs). The lack of overlap in CIs indicated a marked difference in performance. The differences in AUCs and standard error of the difference in AUCs [6, 21] were calculated per paper and utilized in a meta-analysis with the ‘metafor’ [20] package.

We also conducted stratified analysis by operation, continent and time. All ES2 papers were published after 2011; however, we separated the papers into studies solely reporting on patients operated on in or after 2010 (‘post-2010’) and those that contained data on patients operated on prior to 2010 (‘pre-2010’), on whom the authors had retrospectively calculated the ES2. We repeated the main comparisons stratifying by risk of bias (Supplementary Material, Figs. S1–S4). The presence of small-study effects was verified by visual inspection of the funnel plots (Supplementary Material, Figs. S5 and S6). Statistical heterogeneity was tested using Cochrane *Q*-test, and extent of statistical consistency was measured with *I*^2^, which describes the percentage of the variability in effect estimates due to heterogeneity rather than sampling error (chance).

## RESULTS

### Study characteristics

A total of 41 studies published between 2004 and 2020 were included the final analysis. The study characteristics are summarized in Table 1. They contained a heterogenous mix of patients, procedures and locations, commonly found in these studies [6, 22, 23]. Twenty studies reported on all operations performed [2, 24–42], 11 reported on aortic valve replacements with or without coronary artery bypass grafts (CABG) [43–53], 8 CABG only [54–61], 2 on mitral valve repair/replacement [62, 63], 2 on unspecified valvular operations [64, 65] and 1 on thoracic aortic [66] operations. A total of 23 were based in Europe [2, 24, 25, 28, 31, 35–39, 42, 46, 48–50, 53–57, 59, 62, 67], 5 in North America (NA) [32, 41, 44, 58, 63], 4 in South America (SA) [26, 30, 34, 47], 8 in Asia [27, 29, 33, 51, 60, 64–66] and 3 in New Zealand (NZ) [40, 52, 61].

**Table 1:** Overview of study characteristics

Author, year Country	Study period	Sample size	Missing data	Age (years), mean ± SD	Male (%)	Urgency (%)	Case mix (%)	Observed mortality, % (*n*)	Expected mortality	O:E	AUC
Basraon *et al.*, 2011 [44] USA, 1 centre RS	1997–2008	537	NR	70 ± 10	100	Emergency 0.1%	AVR (56% also CABG)	5.9 (32)	**STS** 3.6%	**STS** 1.64	**STS** 0.73
Poullis *et al.*, 2014 [24] **Patients <70 years** Liverpool, UK RS	2006–2010	2437	RF presumed absent	Median 60 SD 4.1	79.5	Urgent 17.8%	CABG 68.2% AVR 53.4%	1.6 (39)	**ES2** 2.5%	**ES2** 0.64	**ES2** 0.80
Poullis *et al.*, 2014 [24] **Patients ≥70 years** Liverpool, UK RS	2006–2010	2147	RF presumed absent	Median 76.4 SD 4.6	65.8	Urgent 21.8%	CABG 31.8% AVR 46.6%	4.3 (92)	**ES2** 5.0%	**ES2** 0.86	**ES2** 0.75
Nashef *et al.*, 2012 [2] 43 European countries, 154 centres PS	May–July 2010	22 381	<1%	64.7 ± 12.5	69.1	Urgent 18.5% Emergency 4.3% Salvage 0.5%	CABG 46.7% Valves 46.3%	3.9 (873)	**ES2** 3.95%	**ES2** 0.99	
Grant *et al.*, 2012 [35] UK Database RS	2010–2011	23 740	Imputation	67.1 ± 11.8	72.3	Urgent 28.7% Emergency 2.9% Salvage 0.3%	CABG 52.5% Valves 21% AVR + CABG 10% Aortic 4.3%	3.1 (736)	**ES2** 3.4%	**ES2** 0.92	**ES2** 0.81
Chalmers *et al.*, 2013 [36] Liverpool, UK RS	2006–2010	5576	RF presumed absent	Median 69.3 SD 10	73.9	Urgent 28.3%	CABG 52.2% AVR + CABG 9.3% Isolated valves 20.7% Aortic 6.2%	2.2 (101)	**ES2** 2.0	**ES2** 1.1	**ES2** 0.79
Di Dedda *et al.*, 2013 [37] Italy, 1 centre RS	2010–2011	1090	NR	64.5 ± 13.5	68.3	Urgent 2.2% Emergency 1.7%	CABG 34.1% Isolated valves 37.2% Aortic 7.8%	3.75 (41)	**ES2** 3.1%	**ES2** 1.2	**ES2** 0.81
Howell *et al.*, 2013 [38] **High-risk patients (ES > 10)** Netherlands and Birmingham RS	2006–2011	933	Nil	Median 74.3 SD 7.7	57.5	Urgent 50.2% Emergency 9.2% Salvage 0.3%	CABG 48.8% 2 procedures 32.6% 3 procedures 18.5%	9.7 (90)	**ES2** 9.3%	**ES2** 1.04	**ES2** 0.67
Biancari *et al.*, 2012 [54] Finland, 1 centre RS	2006–2011	1027	Excluded prior to analysis	67 ± 9.4	77.8	Urgent 45.9% Emergency 8.8%	Isolated CABG	3.7 (38)	**ES2** 4.5%	**ES2** 0.82	**ES2** 0.852
Hogervorst *et al.*, 2018 [55] Netherlands, 1 centre RS	2012–2014	2296	Nil	Median 71 SD 9.6	71.2	Emergency 11.4%	CABG 46.1% OPCAB 6.1%	2.4 (55)	**ES2** 1.6%	**ES2** 1.5	**ES2** 0.871
Provenchère *et al.*, 2017 [39] **Octogenarians** France, 1 centre RS	2006–2012	7161	NR	63 ± 14	68	Urgent 5.7%	CABG 37% Valves 57.7%	5.67 (406)	**ES2** 5.17%	**ES2** 1.1	**ES2** 0.80
Singh *et al.*, 2019 [40] NZ, 1 centre PS	2014–2017	1666	NR	65 ± 11	76	Urgent 32.3% Aortic 9.4%	CABG 56%	1.56 (26)	**ES2** 2.97%	**ES2** 0.53	**ES2** 0.831
Ad *et al.*, 2007 [41] USA, 1 centre **Female patients** RS	2001–2004	692 of 3125	NR	65.8	0	NR	Isolated CABG	2.9 (20)	**STS** 2.6%	**STS** 1.1	**STS** 0.82
Ad *et al.*, 2007 [41] USA, 1 centre **Male patients** RS	2001–2004	2433 of 3125	NR	62.6	100	NR	Isolated CABG	1.5 (37)	**STS** 2.1%	**STS** 0.71	**STS** 0.85
Barili *et al.*, 2013 [46] Italy, 3 centres PS	2006–2012	1758	<1%; multiple imputation	69.8 ± 13.2	55	Urgent 2% Emergency 0%	Isolated AVR	1.4 (25)	**ES2** 1.88% **STS** 2.0%	**ES2** 0.74 **STS** 0.7	**ES2** 0.81 **STS** 0.85
Barili *et al.*, 2014 [42] **Elective** Italy, 3 centres PS	2006–2012	12 201 of 13 871	<1%; multiple imputation	67.3 ± 11.8	68	NR	CABG 51% AVR 39% MVR 26% 2+ procedures 34%	1.7 (210)	**ES2** 2.5%	**ES2** 0.68	**ES2** 0.80
Barili *et al.*, 2014 [42] **Non-elective** Italy, 3 centres PS	2006–2012	1670 of 13 871	<1%; multiple imputation	68.1 ± 11.4	74	NR	CABG 73% AVR 17% MVR14% 2+ procedures 25%	8.1 (125)	**ES2** 6.2%	**ES2** 1.3	**ES2** 0.82
Carnero-Alcázar *et al.*, 2013 [25] Spain, 1 centre PS	2005–2010	3798 of 4780	Excluded patients with missing data	67 ± 10.15	62.3	Emergency 4.63%	CABG 32.4%	5.7 (215)	**ES2** 4.46%	**ES2** 1.27	**ES2** 0.85
Borracci *et al.*, 2014 [26] Argentina, 1 centre PS	2012–2013	503	NR	66.4 ± 10.3	74.8	Urgent or emergency 15.9%	CABG 54.3% Valve 27% Valve + CABG 11.7%	4.17 (21)	**ES2** 3.18%	**ES2** 1.31	**ES2** 0.856
Carosella *et al.*, 2014 [47] Argentina, 4 centres RS	2008–2012	250	NR	68.6 ± 13.3	63.2	Urgent 7.6%	Isolated AVR 67.2% AVR + CABG 32.8%	3.6 (9)	**ES2** 1.64%	**ES2** 2.20	**ES2** 0.76
Chan *et al.*, 2014 [63] Canada, 1 centre RS	2001–2011	1154	NR	63.3	58.8	NR	MVR 73.7% repair - 26.3% replacement	1 (11)	**ES2** 3.0% **STS** 2.3%	**ES2** 0.33 **STS** 0.42	**ES2** 0.67 **STS** 0.74
Nishida *et al.*, 2014 [66] Japan, 1 centre RS	1993–2013	461	NR	63.5 ± 0.7	65	Emergency 35.4%	Thoracic aortic surgery	7.2 (33)	**ES2** 7.4%	**ES2** 0.97	**ES2** 0.770
Paparella *et al.*, 2014 [56] Italy, 7 centres RS	2011–2012	6293	1.6%; replaced with mean values	67.3 ± 11.2	65.9	Urgent 15.1 Emergency 3.9%	Isolated CABG	4.9 (305)	**ES2** 4.4%	**ES2** 1.10	**ES2** 0.83
Spiliopoulos *et al.*, 2014 [53] Germany, 1 centre RS	1999–2005	222	NR	66.16	72.7	NR	AVR + CABG	6.3 (14)	**ES2** 3.99%	**ES2** 1.58	**ES2** 0.77
Garcia-Valentin [67] *et al.*, 2016 Spain, 20 centres RS	2012–2013	4034	Nil	66.6 ± 12.3	63.8	Urgent 39.2% Emergency 4.5%	CABG 25.4%	6.5 (262)	**ES2** 5.7%	**ES2** 1.14	**ES2** 0.79
Kar *et al.*, 2017 [27] India, 1 centre RS	2011–2012	911	Excluded prior to analysis (61)	49.37 ± 13.4	66.5	Urgent 13.5% Emergency 4.7%	No OPCAB CABG 47.8% Valve 46.8% Valve + CABG 5.4%	5.7 (52)	**ES2** 2.9%	**ES2** 1.97	**ES2** 0.76
Kirmani *et al.*, 2013 [28] Liverpool, UK RS	2001–2010	14 432	RF presumed absent	65.3 ± 11	72.4	Urgent 16.5% Emergency 2.2%	CABG 61.7% Valve 26.3% Valve + CABG 12%	3.1 (447)	**ES2** 2.44% **STS** 2.40%	**ES2** 1.27 **STS** 1.29	**ES2** 0.816 **STS** 0.810
Borde *et al.*, 2013 [29] India, 1 centre PS	2011–2012	498	Excluded prior to analysis (39)	60.48 ± 7.51	80.1	Emergency 1.6%	CABG 86.5% AVR 5.2%	1.6 (8)	**ES2** 2.01% **STS** 1.6%	**ES2** 0.80 **STS** 1.0	**ES2** 0.69 **STS** 0.65
Kunt *et al.*, 2013 [57] Turkey, 1 centre RS	2004–2012	428	Nil	74.5 ± 3.9	65	Emergency 3.7%	Isolated CABG	7.9 (34)	**ES2** 1.7% **STS** 5.8%	**ES2** 4.65 **STS** 1.36	**ES2** 0.72 **STS** 0.62
Laurent *et al.*, 2013 [48] France, 1 centre PS	2009–2011	314	Nil	73.4 ± 9.7 (29% ≥80 years)	59	Emergency 3%	Severe AS	5.7 (18)	**ES2** 2.3% **STS** 2.8%	**ES2** 2.48 **STS** 2.04	**ES2** 0.77 **STS** 0.73
Luc *et al.*, 2017 [58] **Patient >80 years** Canada, 1 centre RS	2002–2008	304	RF presumed absent	82.1	74.3	Emergency 3.9%	Isolated CABG	2 (6)	**ES2** 4% **STS** 3%	**ES2** 0.50 **STS** 0.67	**ES2** 0.794 **STS** 0.671
Luc *et al.*, 2017 [58] **Patient ≤80 years** Canada, 1 centre RS	2002–2008	608	RF presumed absent	63.8	84.9	Emergency 2.6%	Isolated CABG	1 (6)	**ES2** 2% **STS** 1%	**ES2** 0.50 **STS** 1.0	**ES2** 0.845 **STS** 0.829
Vilca Mejia *et al.*, 2020 [30] Brazil, 11 centres RS	2013–2017	5222	Imputation	60.6 ± 12	63.6	Urgent 29% Emergency 59.6%	CABG 60.2% AVR 22.3% Aortic 0.82%	7.64 (399)	**ES2** 3.1% **STS** 1.0%	**ES2** 2.46 **STS** 7.64	**ES2** 0.763 **STS** 0.766
Nilsson *et al.*, 2004 [59] Sweden, 1 centre RS	1996–2001	4497	NR	66.4 ± 9.3	77	Urgent 25.1% Emergency 7.2% Salvage 1%	Isolated CABG	1.89 (85)	**STS** 1.89%	**STS** 1.0	**STS** 0.71
Osnabrugge *et al.*, 2014 [32] USA, multicentre RS	2003–2012	50 588	RF presumed absent	64.7 ± 11.2	71.1	NR	CABG 80.8% AVR 8.1%	2.1 (1071)	**ES2** 3.1% **STS** 2.7%	**ES2** 0.68 **STS** 0.78	**ES2** 0.77 **STS** 0.81
Qadir *et al.*, 2014 [60] Pakistan, 1 centre RS	2006–2010	2004	RF presumed absent	58.3 ± 9.6	82.7	Urgent 11.1% Emergency 11.1% Salvage 5.6%	Isolated CABG	3.8 (76)	**ES2** 3.72%	**ES2** 1.02	**ES2** 0.836
Rabbani *et al.*, 2014 [64] Pakistan, 1 centre RS	2006–2013	576 STS: 490	RF presumed absent	47.36 ± 15.5	53.5	NR	Valve replacement surgery ± CABG	5.7 (28)	**ES2** 4.94% **STS** 2.13%	**ES2** 1.15 **STS** 2.68	**ES2** 0.816 **STS** 0.812
Shapira-Daniels *et al.*, 2020 [33] Israel, 1 centre RS	2008–2015	1279	NR	64 ± 12	73	Urgent 47% Emergent/salvage 1%	CABG 62% AVR 17%	1.95 (25)	**ES2** 3.31% **STS** 3.12%	**ES2** 0.59 **STS** 0.63	**ES2** 0.81 **STS** 0.83
Tiveron *et al.*, 2015 [34] Brazil, 1 centre PS	2011–2013	562	NR	NR	NR	NR	CABG 65.5% Valve 28.5% Valve + CABG 6%	4.6 (26)	**ES2** 1.3% **STS** 3.7%	**ES2** 3.54 **STS** 1.24	**ES2** 0.704 **STS** 0.649
Tralhão *et al.*, 2015 [49] **Patients >80 years** Portugal, 1 centre RS	2003–2010	106	RF presumed absent	83.1 ± 2.2	36.8	Urgent 9.4% Emergency 0%	Isolated AVR	5.7 (6)	**ES2** 4.4% **STS** 4.0%	**ES2** 1.30 **STS** 1.43	**ES2** 0.792 **STS** 0.702
Wang *et al.*, 2013 [65] China, 1 centre RS	2006–2011	3479	Imputation	50 ± 12.4	46.2	NR	Valve surgery only	3.2 (112)	**ES2** 2.52% **STS** 3.28%	**ES2** 1.28 **STS** 0.98	**ES2** 0.693 **STS** 0.706
Wang *et al.*, 2014 [61] NZ, 1 centre RS	2010–2012	818	NR	64.5 ± 10.0	79.8	NR	Isolated CABG	1.6 (13)	**ES2** 1.6% **STS** 2.3%	**ES2** 1.0 **STS** 0.70	**ES2** 0.642 **STS** 0.641
Wang *et al.*, 2015 [52] NZ, 1 centre RS	2005–2012	620	NR	64.8 ± 15.5	65.5	Urgent 50.6% Emergency 0.3%	AVR ± CABG	2.9 (18)	**ES2** 3.8% **STS** 2.8%	**ES2** 0.76 **STS** 1.04	**ES2** 0.711 **STS** 0.684
Wendt *et al.*, 2014 [50] Germany, 1 centre RS	1999–2012	1066	Nil	68.3 ± 11.5	53.8	NR	AVR ± CABG	4.2 (45)	**ES2** 3.2% **STS** 4.8%	**ES2** 1.31 **STS** 0.88	**ES2** 0.724 **STS** 0.726
Yamaoka *et al.*, 2016 [51] Japan, 1 centre RS	2002–2013	406	NR	71.6 ± 9.9	53	Urgent/emergency 2%	AVR ± CABG	3.4 (14)	**ES2** 3.1% **STS** 4.9%	**ES2** 1.09 **STS** 0.69	**ES2** 0.704 **STS** 0.781

Bold representation is to highlight the different patient populations AUC: area under the receiver operator curve; AVR: aortic valve replacement; CABG: coronary artery bypass graft; ES: European System for Cardiac Operative Risk Evaluation; MVR: mitral valve repair/replacement; NR: not reported; NZ: New Zealand; O:E: observed-to-expected mortality; PS: prospective; RF: risk factor; RS: retrospective; SD: standard deviation; STS: Society of Thoracic Surgeons.

The necessary data could be derived from 39 studies [2, 24–30, 32–34, 36–40, 42, 46–58, 60–68] (42 independent populations; 190 378 patients, 6254 deaths) on ES2 and 21 studies [28–30, 32–34, 41, 44, 46, 48–52, 57–59, 63–65] (23 independent populations; 92 291 patients; 2477 deaths) on STS score, 18 papers [28–30, 32–34, 46, 48–52, 57, 58, 61, 63–65] (19 independent populations; 84 132 patients; 3455 deaths) comparing ES2 and STS.

### Individual model performance

#### European System for Cardiac Operative Risk Evaluation 2 in individual studies

**Figure 2:**
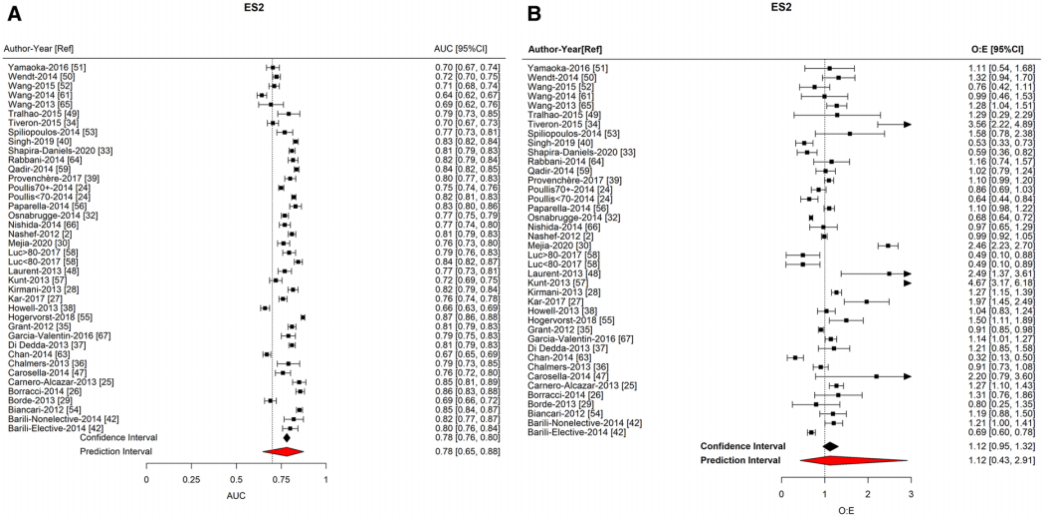
Forest plots of meta-analysis of European System for Cardiac Operative Risk Evaluation 2. (**A**) Area under the receiver operator curve. (**B**) Observed-to-expected ratio.

The ES2 showed good discrimination (AUC = 0.782; 95% CI: 0.763–0.800; 95% PI: 0.646–0.875) and calibration (O:E = 1.118; 95% CI: 0.950–1.317; 95% PI: 0.430–2.912) (Fig. 2/Table 2). There was no significant difference in AUC between studies at high and low risks of bias (Supplementary Material, Figs. S1 and S2), between continents nor between studies reporting on patients operated on before and after 2010 (Supplementary Material, Fig. S7).

**Table 2:** Tabulated results of meta-analyses

Prediction model	Parameter measured	Number of studies	Summary	95% CI	95% PI	*I* ^2^
Individual model performance						
ES2	Discrimination (AUC)	40	0.782	0.763 to 0.800	0.646 to 0.875	95.4
	Calibration (O:E)	40	1.118	0.950 to 1.317	0.430 to 2.912	97.0
STS	Discrimination (AUC)	23	0.757	0.727 to 0.785	0.651 to 0.839	56.4
	Calibration (O:E)	23	1.111	0.853 to 1.447	0.0.318 to 3.889	96.8

Parameter measured	Prediction model	Number of studies	Summary	95% CI	95% PI	*I* ^2^
Comparison of prediction models						
Discrimination (AUC)	ES2	19	0.756	0.728 to 0.783	0.623 to 0.854	94.6
	STS	19	0.752	0.720 to 0.781	0.638 to 0.839	60.8
	Difference	19	−0.016	−0.034 to 0.002	−0.035 to 0.004	
Calibration (O:E)	ES2	19	1.124	0.804 to 1.71	0.271 to 4.664	97.6
	STS	19	1.116	0.812 to 1.535	0.279 to 4.470	97.5

AUC: area under the receiver operator curve; CI: confidence interval; ES2: European System for Cardiac Operative Risk Evaluation 2; O:E: observed-to-expected mortality ratio; PI: prediction interval; STS: Society of Thoracic Surgeons.

We found that ES2 calibration varied significantly between continents (*P* < 0.0001). ES2 overestimated risk in NA (O:E = 0.515; 95% CI: 0.312–0.718) and NZ (O:E = 0.680; 95% CI: 0.429–0.931) and under-estimated risk in SA (O:E = 2.279; 95% CI: 1.403–3.155). ES2 had a trend towards risk underestimation in ‘post-2010’ studies (O:E = 1.368; 95% CI: 1.004–1.732) compared to ‘pre-2010’ studies (O:E = 0.991; 95% CI: 0.854–1.128)(*P* = 0.057) (Table 3/Supplementary Material, Fig. S8). There was statistical evidence of an association between AUC and O:E and the type of operation (*P* < 0.0001), largely driven by in 1 mitral study (Table 3).

**Table 3:** Subgroup analysis of European System for Cardiac Operative Risk Evaluation 2


	Number of studies	Summary	CI	*I* ^2^
Discrimination (AUC)				
Summary estimate	40	0.782	0.763–0.800	95.4
Subgroup analysis				
By operation (all studies: *P* < 0.0001; excluding MVR: *P* = 0.07)				
AVR ± CABG	7	0.742	0.718–0.766	64.5
CABG	7	0.789	0.730–0.848	97.4
MVR	1	0.670	0.648–0.692	–
Valve	2	0.759	0.639–0.879	90.5
Mixed	22	0.790	0.768–0.813	95.8
Aortic	1	0.759	0.739–0.879	–
By continent (*P* = 0.557)				
Europe	21	0.793	0.771–0.815	95.6
North America	4	0.770	0.697–0.842	97.6
South America	4	0.771	0.708–0.835	95.3
Asia	8	0.763	0.4723–0.803	94.6
NZ	3	0.729	0.620–0.837	98.9
Studies containing patients operated on prior to 2010 (*P* = 0.397)				
Pre-2010	28	0.772	0.751–0.793	95.3
Post-2010	12	0.790	0.754–0.827	97
Calibration (O:E)				
Summary estimate	40	1.118	0.950–1.317	97.0
Subgroup analysis				
By operation (all studies: *P* < 0.0001; excluding MVR: *P* = 0.55)				
AVR ± CABG	7	1.335	0.950–1.721	58.2
CABG	7	1.267	0.449–2.086	84.7
MVR	1	0.318	0.131–0.515	–
Valve	2	1.249	1.046–1.452	0
Mixed	22	1.126	0.918–1.334	95.6
Aortic	1	0.967	0.649–1.285	–
By continent (*P* < 0.0001)				
Europe	21	1.099	0.987–1.211	87.2
North America	5	0.515	0.312–0.718	80.6
South America	4	2.279	1.403–3.155	83.1
Asia	8	1.087	0.824–1.350	78.3
NZ	3	0.680	0.429–0.931	40.8
Studies containing patients operated on prior to 2010 (*P* = 0.057)				
Pre-2010	28	0.991	0.854–1.128	91
Post-2010	12	1.368	1.004–1.732	95.1

AUC: area under the receiver operator curve; AVR: aortic valve replacement; CABG: coronary artery bypass graft; CI: confidence interval; MVR: mitral valve repair/replacement; NZ: New Zealand; O:E: observed-to-expected mortality ratio.

#### Society of Thoracic Surgeons in individual studies

**Figure 3:**
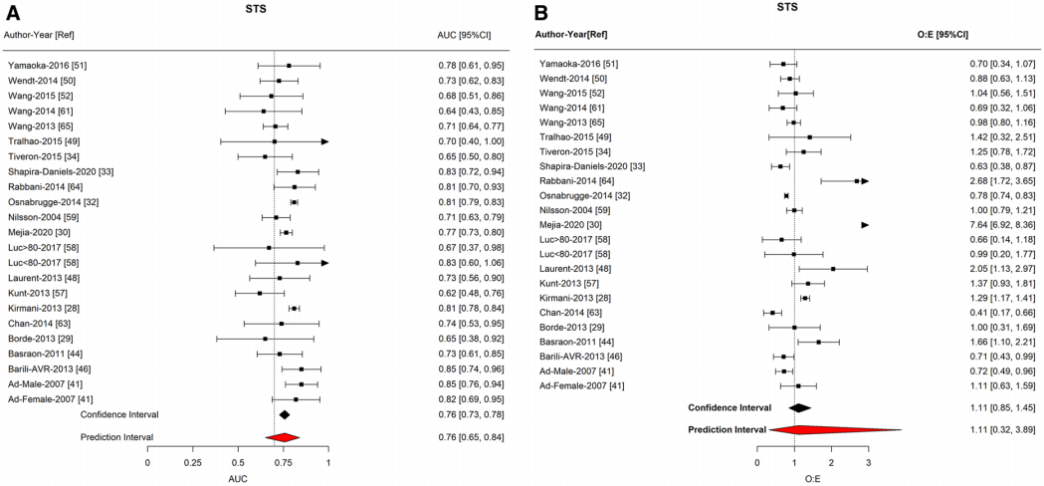
Forest plots of meta-analysis of Society of Thoracic Surgeons score. (**A**) Area under the receiver operator curve. (**B**) Observed-to-expected ratio.

STS demonstrated good discrimination (AUC = 0.757; 95% CI: 0.727–0.785; 95% PI: 0.651–0.839) and calibration (O:E = 1.111; 95% CI: 0.853–1.447; 95% PI: 0.318–3.889; Fig. 3/Table 2). There was a statistically significant correlation between AUC and the continent of the study (*P* = 0.03; Table 4/Supplementary Material, Fig. S9), with the lower extent of CIs falling noticeably below 0.7 for SA (0.731; 95% CI: 0.627–0.834) and NZ (0.667; 95% CI: 0.532–0.801). There was strong statistical evidence of an association between calibration and operation (*P* = 0.0018), largely driven by in 1 mitral study (Table 4). There were no significant differences in STS score between continents nor over time.

**Table 4:** Subgroup analysis of Society of Thoracic Surgeons


	Number of studies	Summary	CI	*I* ^2^
Discrimination (AUC)				
Summary estimate	23	0.757	0.727 to 0.785	56.4
Subgroup analysis				
By operation (all studies: *P* = 0.22; excluding MVR: *P* = 0.13)				
AVR ± CABG	6	0.728	0.667 to 0.789	0
CABG	7	0.745	0.772 to 0.821	51
MVR	1	0.740	0.533 to 0.947	–
Valve	2	0.749	0.647 to 0.851	58.9
Mixed	7	0.797	0.772 to 0.821	48.6
Aortic	0	–	–	–
By continent (*P* = 0.03)				
Europe	6	0.751	0.684 to 0.818	66.6
North America	7	0.809	0.792 to 0.827	0
South America	2	0.731	0.627 to 0.836	55
Asia	6	0.758	0.699 to 0.817	6
NZ	2	0.667	0.532 to 0.801	0
Studies containing patients operated on prior to 2010 (*P* = 0.21)				
Pre-2010	19	0.773	0.742 to 0.805	40.6
Post-2010	4	0.714	0.628 to 0.801	25.4
Calibration (O:E)				
Summary estimate	23	1.111	0.853 to 1.447	96.8
Subgroup analysis				
By operation (all studies: *P* = 0.0018; excluding MVR: *P* = 0.36)				
AVR ± CABG	6	1.171	0.788 to 1.555	65.1
CABG	7	0.913	0.726 to 1.100	41.5
MVR	1	0.414	0.171 to 0.658	–
Valve	2	1.763	0.102 to 3.425	91.3
Mixed	7	1.888	0.024 to 3.752	98.5
Aortic	0	–	–	–
By continent (*P* = 0.42)				
Europe	6	1.056	0.832 to 1.279	77.9
North America	7	0.847	0.573 to 1.122	71
South America	2	4.440	−1.823 to 10.702	99.5
Asia	6	1.230	0.640 to 1.820	80.8
NZ	2	0.832	0.499 to 1.166	21.3
Studies containing patients operated on prior to 2010 (*P* = 0.37)				
Pre-2010	19	0.987	0.815 to 1.159	85.1
Post-2010	4	2.639	−0.622 to 5.901	99

AUC: area under the receiver operator curve; AVR: aortic valve replacement; CABG: coronary artery bypass graft; CI: confidence interval; MVR: mitral valve repair/replacement; NZ: New Zealand; O:E: observed-to-expected mortality ratio.

#### European System for Cardiac Operative Risk Evaluation 2 versus Society of Thoracic Surgeons in comparative studies

**Figure 4:**
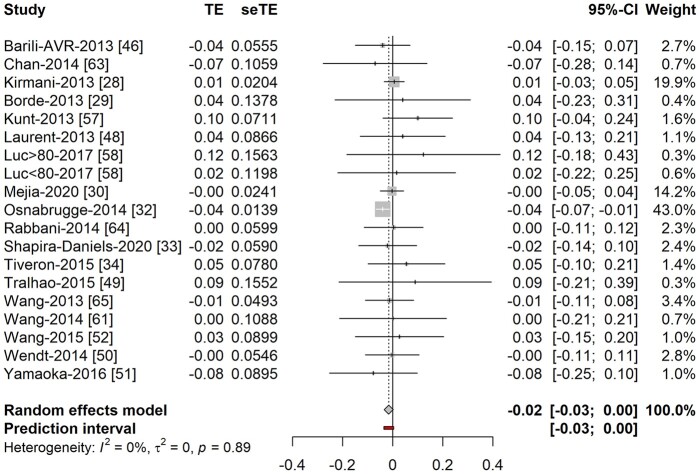
Difference in discrimination of European System for Cardiac Operative Risk Evaluation 2 and Society of Thoracic Surgeons score. TE: difference in *C*-stastistic; seTE: standard error of difference in *C*-statistic.

There was no difference in discrimination between ES2 [AUC: 0.756 (95% CI: 0.728–0.783)] and STS [AUC: 0.752 (95% CI: 0.720–0.781)], with no statistically significant difference in the AUC [−0.016 (95% CI: −0.033 to 0.002); *P* = 0.9; Table 2/Fig. 4]. The pooled estimates of the O:E for the ES2 (1.124; 95% CI: 0.804–1.710) and STS (1.116; 95% CI: 0.812–1.535) were also similar with overlap between their CIs.

## DISCUSSION

We compared the performance of the 2 most used mortality prediction models in adult cardiac surgery-ES2 and STS scores, using measures of discrimination (AUC) and calibration (O:E). Discrimination is a model’s ability to successfully differentiate between those likely and unlikely to experience an event in each population. Calibration describes the certainty with which it can predict the occurrence of an event in an individual. Both should be optimized to have a truly efficient model. Our results build on findings from 3 previous meta-analyses [6, 22, 23] by providing a dedicated statistical technique to quantitatively assess calibration in addition to discrimination and performing extended subgroup analysis.

The most notable finding of our study was that whilst the ES2 and STS performed well across the whole population, there was significant variation in the performance of ES2 between continents. It was shown to work well in the continent from which it was derived (i.e. Europe) but over-predicted risk in NA and NZ and under-predicted risk in SA. The availability of the coefficients for ES2 in the public domain may explain why this is more widely reported and there are substantially more papers from Europe. There was a tendency of ES2 to under-predict risk in papers with patients operated on solely after 2010.

However, the STS score showed good and stable performance in all continents and across both time periods studied. The STS score regression coefficients are not in the public domain and it utilizes far more variables to provide procedure-specific outcome calculations of morbidity and mortality. Consequently, the STS score performance was reported far less frequently. A key difference in the models is that STS is recalibrated annually to ensure the O:E ratio remains around 1 [10, 11].

Analysis of papers providing direct comparisons of calibration of the 2 models suggested a non-significant difference between them. The same predominance of European papers was not seen here and this may account for the discrepancy in our findings. It would have been interesting to evaluate the calibration of these models using the calibration slope or calibration in large, however this is often not reported. The Hosmer–Lemeshow statistic is one of the most widely reported statistics regarding model calibration but does not lend itself to statistical comparison between studies.

Over time the risk profile of patients has increased but operative mortality has decreased and ES has been shown to suffer from poor calibration, especially in those at highest risk [69–73]. The lack of availability of individual patient-level data limited our ability to analyse differential model performance in high and low-risk populations. Further review of these population subgroups would be of clinical importance.

Clinicians need to balance the superior performance of the STS with the relative parsimony and ease of use of ES2. Our findings suggest that ES2 and STS can be used in the populations from which they are derived but that STS may offer advantages when performing comparative research across continents.

### Limitations

Bias may have been introduced into the study as we only reviewed articles in English. Abstracts and unpublished works could not be included and may have resulted in publication bias. Small study effects and significant heterogeneity could not be negated despite performing meta-regression, subgroup and sensitivity analyses. We were only able to compare studies in whom the AUC and O:E ratios could be derived, and a large study [74] was excluded due to this. Reclassification metrics have been shown to be a good estimate of model discrimination [75]; however, they were not reported in these studies and the lack of individual patient-level data made their derivation impossible.

The ES2 and STS calibration demonstrated statistically significant differences by type of operation which was driven by a singular study on mitral operations. Most studies evaluated either a mixed population, aortic valve replacements ± CABG or isolated CABG. There were few studies with dedicated performance measures on mitral valve, aortic or off-pump CABG and so the utility of these scoring systems in these subgroups could not be evaluated accurately. With the increasing number of ‘prophylactic’ aortic aneurysm operations being conducted and the emergence of transcatheter mitral interventions the validation of existing risk prediction models in these populations will become increasingly relevant.

Some interventional cardiologists have reported the use of these scoring systems in the prediction of risk in their patients and this is partially reflected in the latest guidelines [7]. We did not review the accuracy of these models in patients undergoing interventional procedures and so cannot comment on their applicability in this setting.

## CONCLUSIONS

The results of this meta-analysis validate the use of either ES2 or STS in the prediction of mortality following adult cardiac surgery, especially in the continent from which they were derived. Both scores show good discrimination throughout the populations studied. The STS may be better calibrated when evaluating outcomes across European and North American centres. Future research should focus on analysis of large databases of individual patient-level data to corroborate these findings.

## SUPPLEMENTARY MATERIAL

Supplementary material is available at *ICVTS* online.

## Supplementary Material

ivab151_Supplementary_Data

## ABBREVIATIONS

AUC: Area under the receiver operator curve
CI: Confidence interval
CABG: Coronary artery bypass grafts
ES: European System for Cardiac Operative Risk Evaluation
STS: Society of Thoracic Surgeons
NZ: New Zealand
NA: North America
O:E: Observed-to-expected mortality
PI: Prediction interval
SA: South America
