# Supplementary material for: Systematic review and meta-analysis of mortality risk prediction models in adult cardiac surgery
Source: Interact Cardiovasc Thorac Surg. 2021 May 26;33(5):673–86. doi: 10.1093/icvts/ivab151 (PMC8557799; doi:10.1093/icvts/ivab151)
Supplement: ivab151_Supplementary_Data [file ivab151_supplementary_data.docx]

**Supplemental Material**

Systematic Review and Meta-Analysis of Mortality Risk Prediction Models in Adult Cardiac Surgery. Sinha et. al.

**Supplementary Table 1. Search String**

**Search conducted using the NICE Healthcare Databases Advanced Search.**

| **#** | **Database** | **Search term** | **Results** |
| --- | --- | --- | --- |
| 1 | Medline | (((cardi* OR coronary OR heart) ADJ3 surg*) AND (adult* OR patient* OR ((older OR elder*) ADJ2 (people OR person OR adult*)))).ti,ab | 63601 |
| 2 | Medline | (((score OR scoring) ADJ4 (system* OR model*)) AND (mortality ADJ3 predict*)).ti,ab | 2385 |
| 3 | Medline | (1 AND 2) | 151 |
| 4 | Medline | (child*).ti,ab | 1363916 |
| 5 | Medline | 3 NOT 4 | 145 |
| 6 | Medline | (assess* OR compar* OR validation OR predict*).ti,ab | 8257566 |
| 7 | Medline | (5 AND 6) | 145 |
| 8 | CINAHL | (((cardi* OR coronary OR heart) ADJ3 surg*) AND (adult* OR patient* OR ((older OR elder*) ADJ2 (people OR person OR adult*)))).ti,ab | 17265 |
| 9 | CINAHL | (((score OR scoring) ADJ4 (system* OR model*)) AND (mortality ADJ3 predict*)).ti,ab | 753 |
| 10 | CINAHL | (8 AND 9) | 22 |
| 11 | CINAHL | (child*).ti,ab | 481604 |
| 12 | CINAHL | 10 NOT 11 | 22 |
| 13 | CINAHL | (assess* OR compar* OR validation OR predict*).ti,ab | 1693276 |
| 14 | CINAHL | (12 AND 13) | 23 |
| 15 | EMBASE | (((cardi* OR coronary OR heart) ADJ3 surg*) AND (adult* OR patient* OR ((older OR elder*) ADJ2 (people OR person OR adult*)))).ti,ab | 105700 |
| 16 | EMBASE | (((score OR scoring) ADJ4 (system* OR model*)) AND (mortality ADJ3 predict*)).ti,ab | 3858 |
| 17 | EMBASE | (15 AND 16) | 231 |
| 18 | EMBASE | (child*).ti,ab | 1738436 |
| 19 | EMBASE | 17 NOT 18 | 219 |
| 20 | EMBASE | (assess* OR compar* OR validation OR predict*).ti,ab | 11211029 |
| 21 | EMBASE | (19 AND 20) | 222 |
| 22 | EMCARE | (((cardi* OR coronary OR heart) ADJ3 surg*) AND (adult* OR patient* OR ((older OR elder*) ADJ2 (people OR person OR adult*)))).ti,ab | 25193 |
| 23 | EMCARE | (((score OR scoring) ADJ4 (system* OR model*)) AND (mortality ADJ3 predict*)).ti,ab | 927 |
| 24 | EMCARE | (22 AND 23) | 44 |
| 25 | EMCARE | (child*).ti,ab | 518600 |
| 26 | EMCARE | 24 NOT 25 | 42 |
| 27 | EMCARE | (assess* OR compar* OR validation OR predict*).ti,ab | 2452573 |
| 28 | EMCARE | (26 AND 27) | 43 |

**Sources searched:** Medline (1946 to present), CINAHL (1981 to present), EMBASE (1974 to present), and EMCARE (1946 to present).

**Supplementary Table 2. List of Excluded Articles with Rationale.**

| **Paper Excluded** | **Rationale** |
| --- | --- |
| Prine et. al., 2012^1^ | Review Article |
| Granton and Cheng, 2008^2^ | Review Article |
| Stoica et. al., 2002^3^ | Review Article |
| Hickey et. al., 2013^4^ | Review Article |
| Nashef et al., 2013^5^ | Editorial commentary |
| Keogh, 2006^6^ | Editorial commentary |
| Hu et. al., 2020^7^ | Calibration metric (O:E) could not be derived from available results. |
| Barili et. al., 2014^8^ | Overlapping population with another study |
| Goetzenich et. al., 2012^9^ | Reported on TAVI population |
| Barili et. al., 2010^10^ | Reported Log ES results only |
| Barmettler et. al., 2004^11^ | Reported ES1 results only |
| Shanmugam et. al., 2005^12^ | Reported ES1/LogES only |
| Kalavrouziotis et. al., 2009^13^ | Reported Log ES results only |
| Ranucci et. al., 2009^14^ | Report on recalibrated Log ES only |

**Supplementary Table 3. Risk of Bias (RoB) Assessment. + Low RoB, - High RoB, ? Uncertain RoB**

|  | RoB |  |  |  |  |  | Applicability |  | Overall |  |
| --- | --- | --- | --- | --- | --- | --- | --- | --- | --- | --- |
|  | Participants | Predictors | Outcome | Sample Size | Missing Data | Statistical Analysis | Predictors | Outcomes | RoB | Applicability |
| Basraon et. al., 2011^15^ | + | + | + | - | - | + | + | + | - | + |
| Poullis et. al., 2014^16^ | + | + | + | - | + | + | + | - | + | + |
| Nashef et. al., 2012^17^ | + | + | + | + | + | + | + | + | + | + |
| Grant et. al., 2012^18^ | + | + | + | + | + | + | + | + | + | + |
| Chalmers et. al., 2013^19^ | + | + | + | + | + | + | + | + | + | + |
| Di Dedda et. al., 2013^20^ | + | + | + | - | - | ? | + | + | - | + |
| Howell et.al., 2013^21^ | + | + | + | - | - | + | + | + | - | + |
| Biancari et. al., 2012^22^ | + | + | + | - | - | ? | + | + | - | + |
| Hogervorst et. al., 2018^23^ | + | + | + | - | + | + | + | + | - | + |
| Provenchère, 2017^24^ | + | + | + | + | - | + | + | + | + | + |
| Singh et. al., 2019^25^ | + | + | + | - | - | + | + | + | + | + |
| Ad et. al., 2007^26^ | + | + | + | - | ? | + | + | + | - | + |
| Barili et. al., 2013_AVR^27^ | + | + | + | - | + | + | + | + | - | + |
| Barili et. al., 2014_Elective/Non-elective^28^ | + | + | + | + | + | + | + | + | + | + |
| Carnero-Alcázar et. al., 2013^29^ | + | + | + | + | - | + | + | + | + | + |
| Borracci et. al., 2014^30^ | + | + | + | - | - | + | + | + | - | + |
| Carosella et. al, 2014^31^ | + | + | + | - | - | + | + | + | - | + |
| Chan et. al., 2014^32^ | + | + | + | - | - | + | + | + | - | + |
| Nishida et. al., 2014^33^ | + | + | + | - | - | + | + | + | - | + |
| Paparella et. al., 2014^34^ | + | + | + | + | + | + | + | + | + | + |
| Spiliopoulos et. al., 2014^35^ | + | + | + | - | - | + | + | + | - | + |
| Garcia-Valentin et. al., 2016^36^ | + | + | + | + | + | + | + | + | + | + |
| Kar et.al., 2017^37^ | + | + | + | - | - | + | + | + | - | + |
| Kirmani et. al., 2013^38^ | + | + | + | + | + | + | + | + | + | + |
| Borde et. al., 2013^39^ | + | + | + | - | - | + | + | + | - | + |
| Kunt et. al., 2013^40^ | + | + | + | - | + | + | + | + | - | + |
| Laurent et. al., 2013^41^ | + | + | + | - | + | + | + | + | - | + |
| Luc et. al., 2017^42^ | + | + | + | - | + | + | + | + | - | + |
| Mejia et. al, 2020^43^ | + | + | + | + | + | + | + | + | + | + |
| Nilsson et. al., 2004^44^ | + | + | + | - | + | + | + | + | + | + |
| Osnabrugge et. al., 2014^45^ | + | + | + | + | + | + | + | + | + | + |
| Qadir et. al., 2014^46^ | + | + | + | - | + | + | + | + | ? | + |
| Rabbani et. al., 2014^47^ | + | + | + | - | + | + | + | + | - | + |
| Shapira-Daniels et. al., 2020^48^ | + | + | + | - | - | + | + | + | - | + |
| Tiveron et. al., 2015^49^ | + | + | + | - | - | + | + | + | - | + |
| Tralhão et. al., 2015^50^ | + | + | + | - | + | + | + | + | - | + |
| Wang et. al., 2013^51^ | + | + | + | + | + | + | + | + | + | + |
| Wang et. al., 2014^52^ | + | + | + | - | - | + | + | + | - | + |
| Wang et. al., 2015^53^ | + | + | + | - | - | + | + | + | - | + |
| Wendt et. al., 2014^54^ | + | + | + | - | + | + | + | + | ? | + |
| Yamaoka et. al., 2016^55^ | + | + | + | - | - | + | + | + | - | + |

**Supplementary Figure 1. EuroSCORE2.** AUC – stratified by risk of bias.


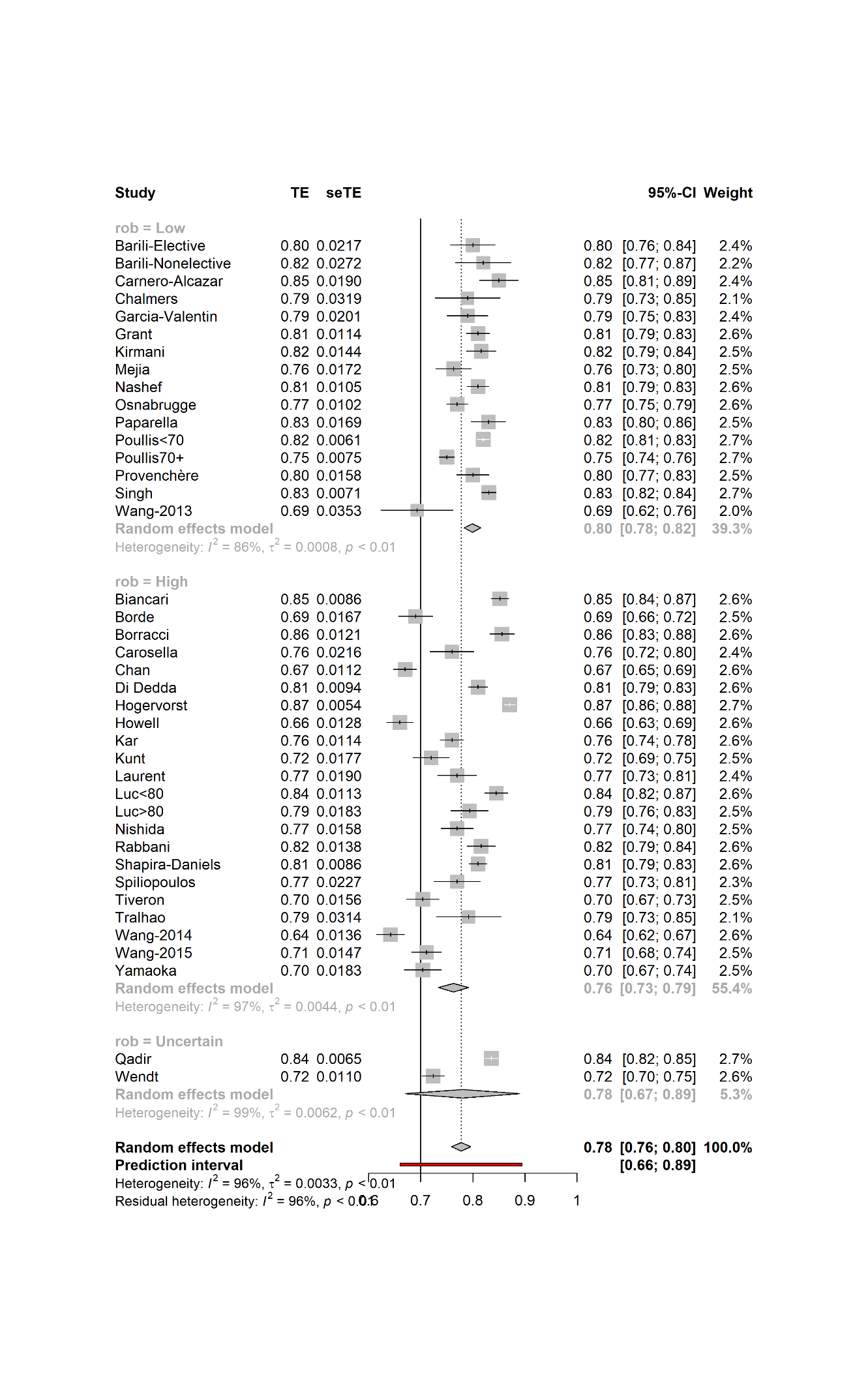


TE: c-statistic. seTE: standard error of the c-statistic.

**Supplementary Figure 2. EuroSCORE2.** O:E – stratified by risk of bias.


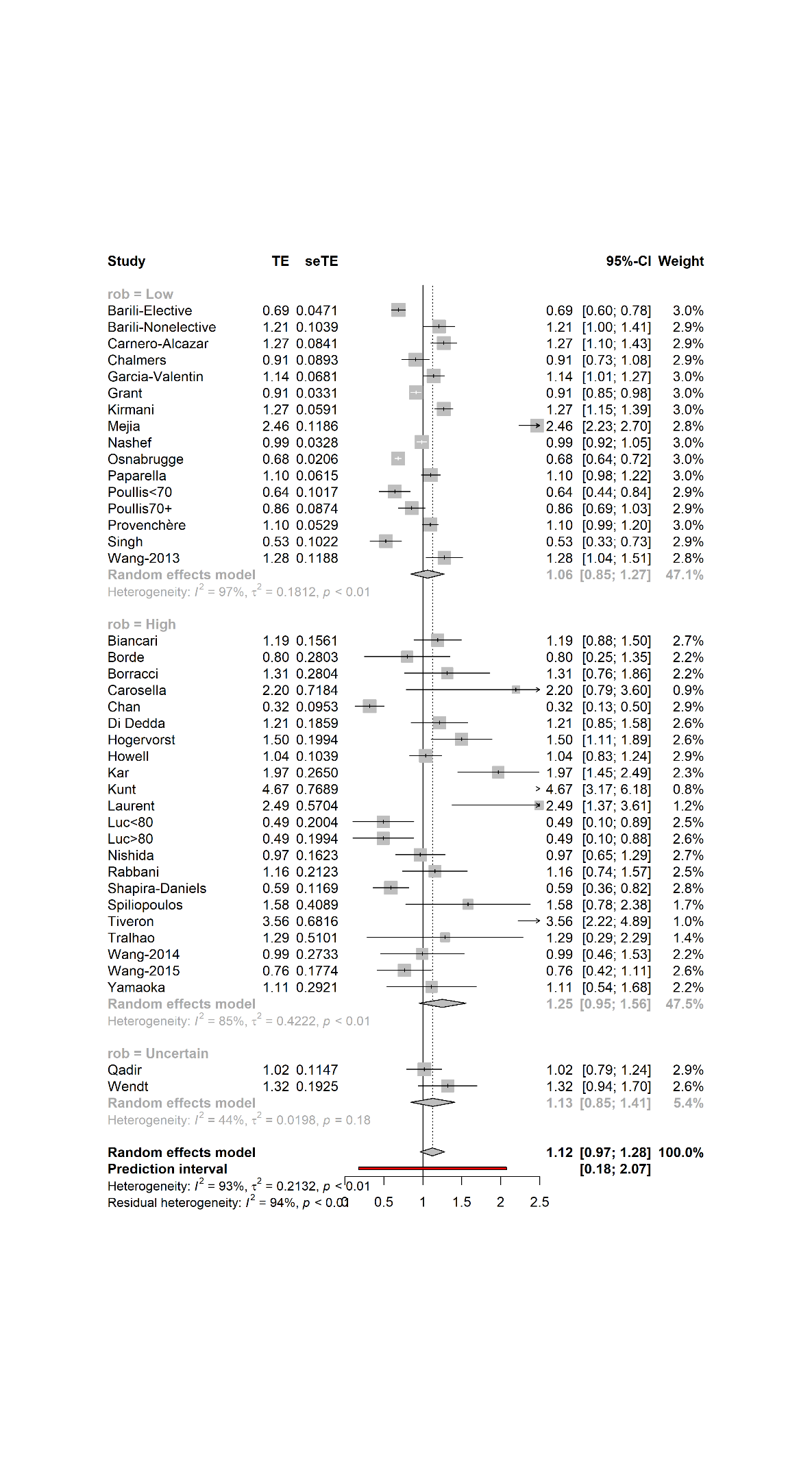


TE: O:E ratio. seTE: standard error of O:E ratio.

**Supplementary Figure 3. Society of Thoracic Surgeons Score.** AUC – stratified by risk of bias.


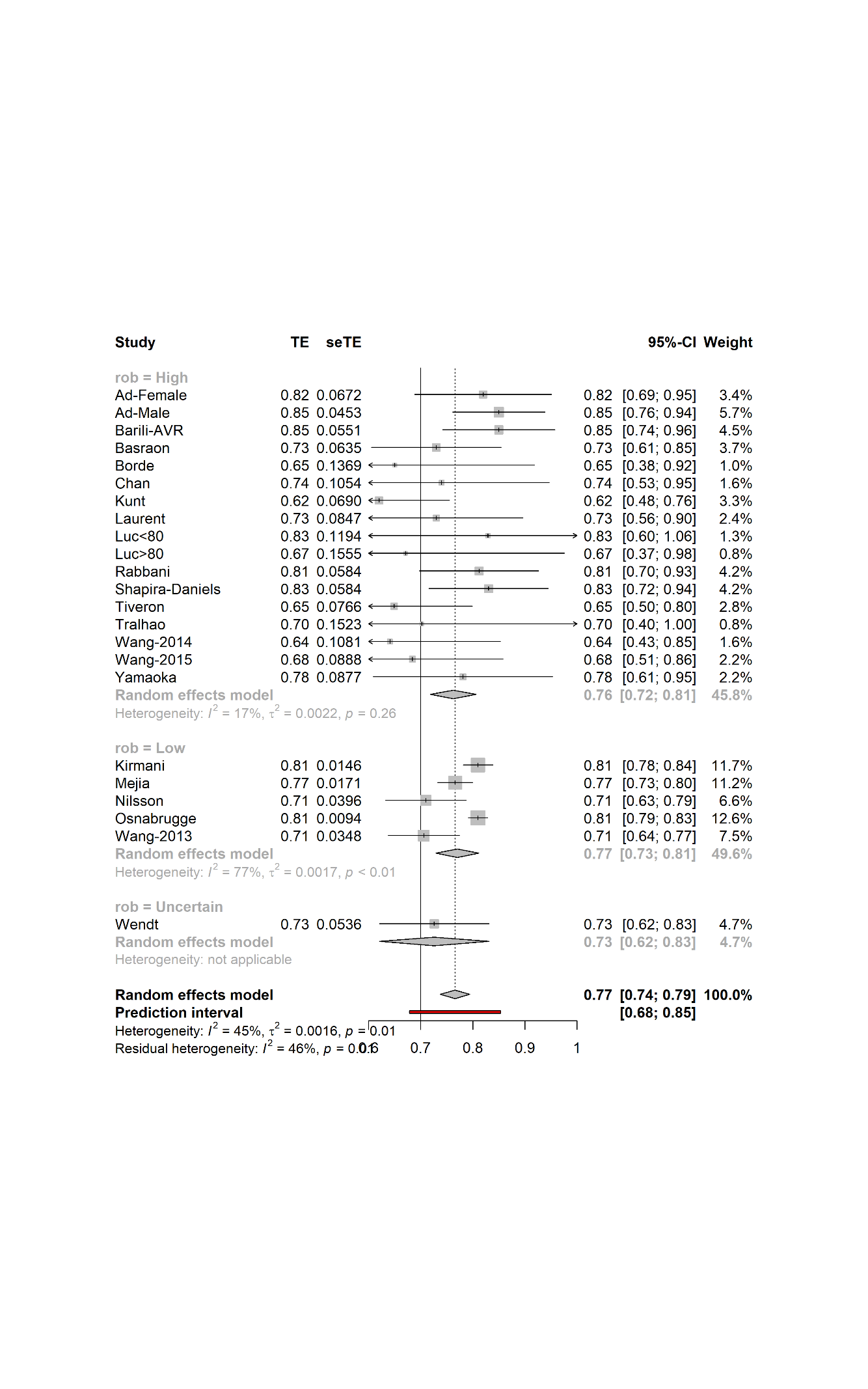


TE: c-statistic. seTE: standard error of the c-statistic.

**Supplementary Figure 4. Society of Thoracic Surgeons Score.** O:E – stratified by risk of bias.


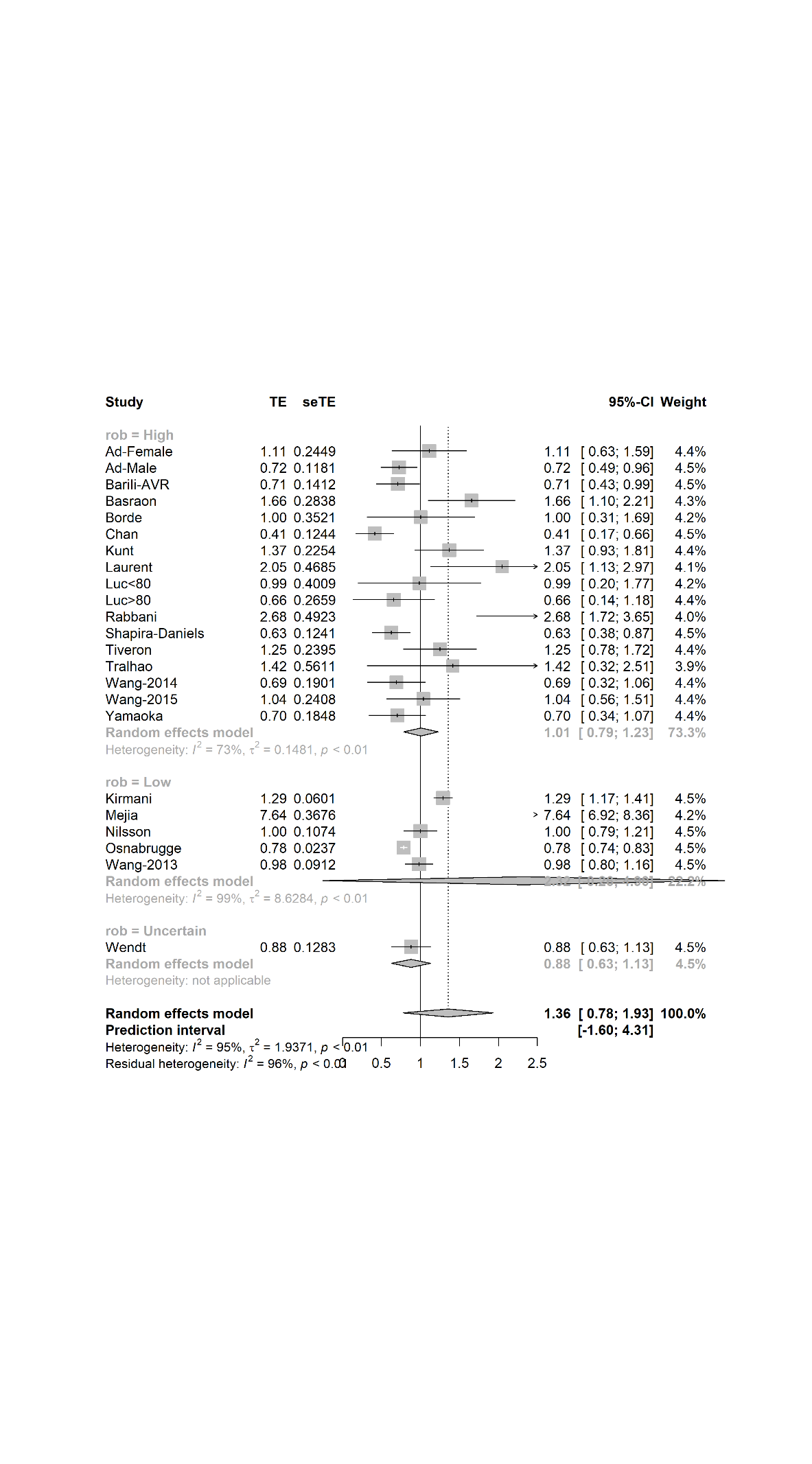


TE: O:E ratio. seTE: standard error of O:E ratio

**Supplementary Figure 5.** Funnel plots of AUC and O:E for EuroSCORE2

**
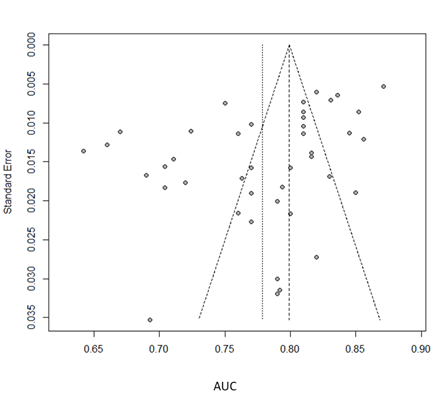
**

**
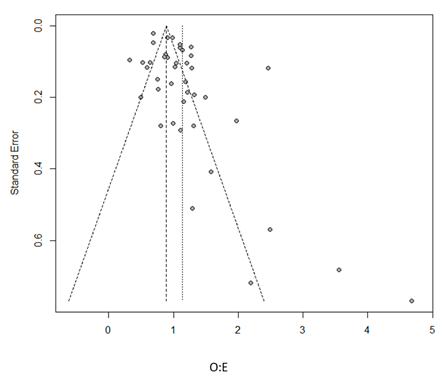
**

**Supplementary Figure 6.** Funnel plots of AUC and O:E for Society of Thoracic Surgeons Score.


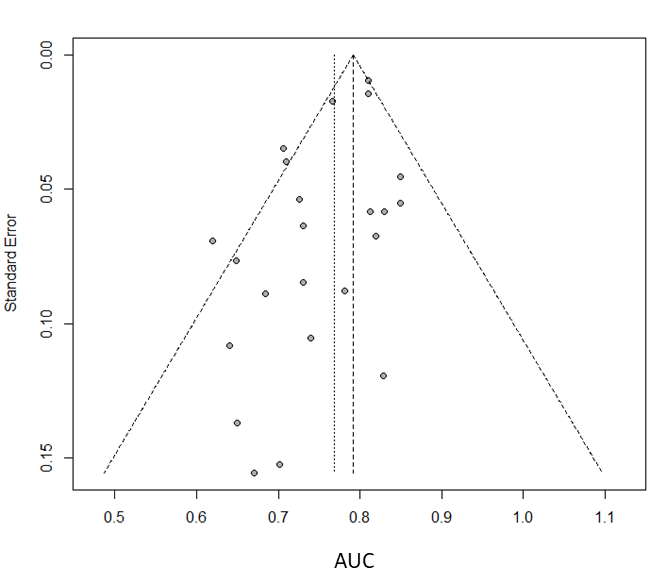


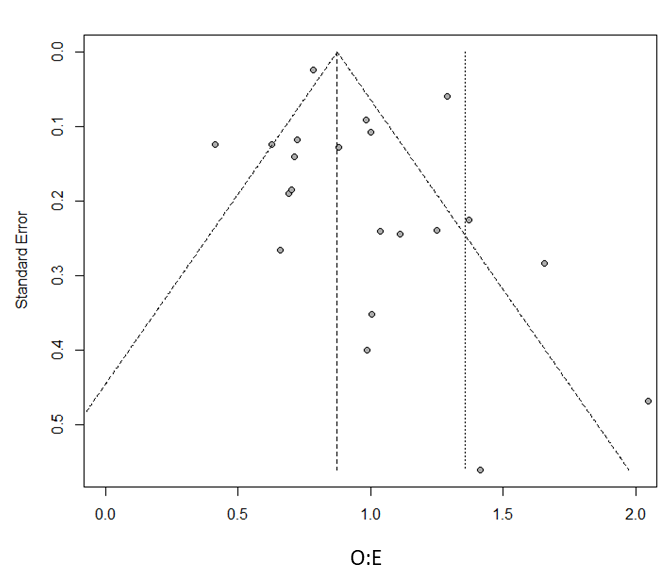


**Supplementary Figure 7. EuroSCORE2 –** Subgroup Analysis of Discrimination. TE: c-statistic. seTE: standard error of the c-statistic

1. By Operation


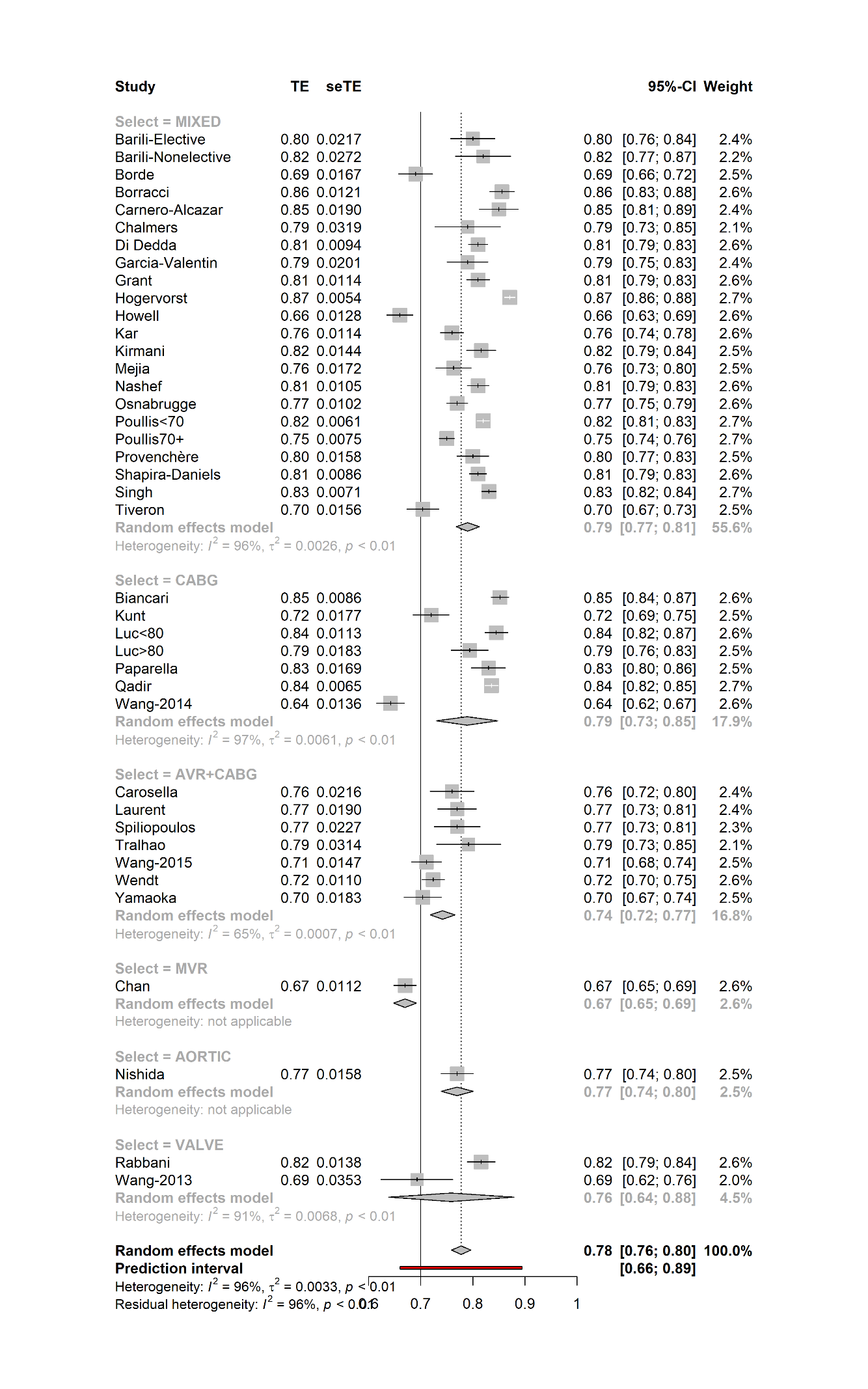


1. By Continent


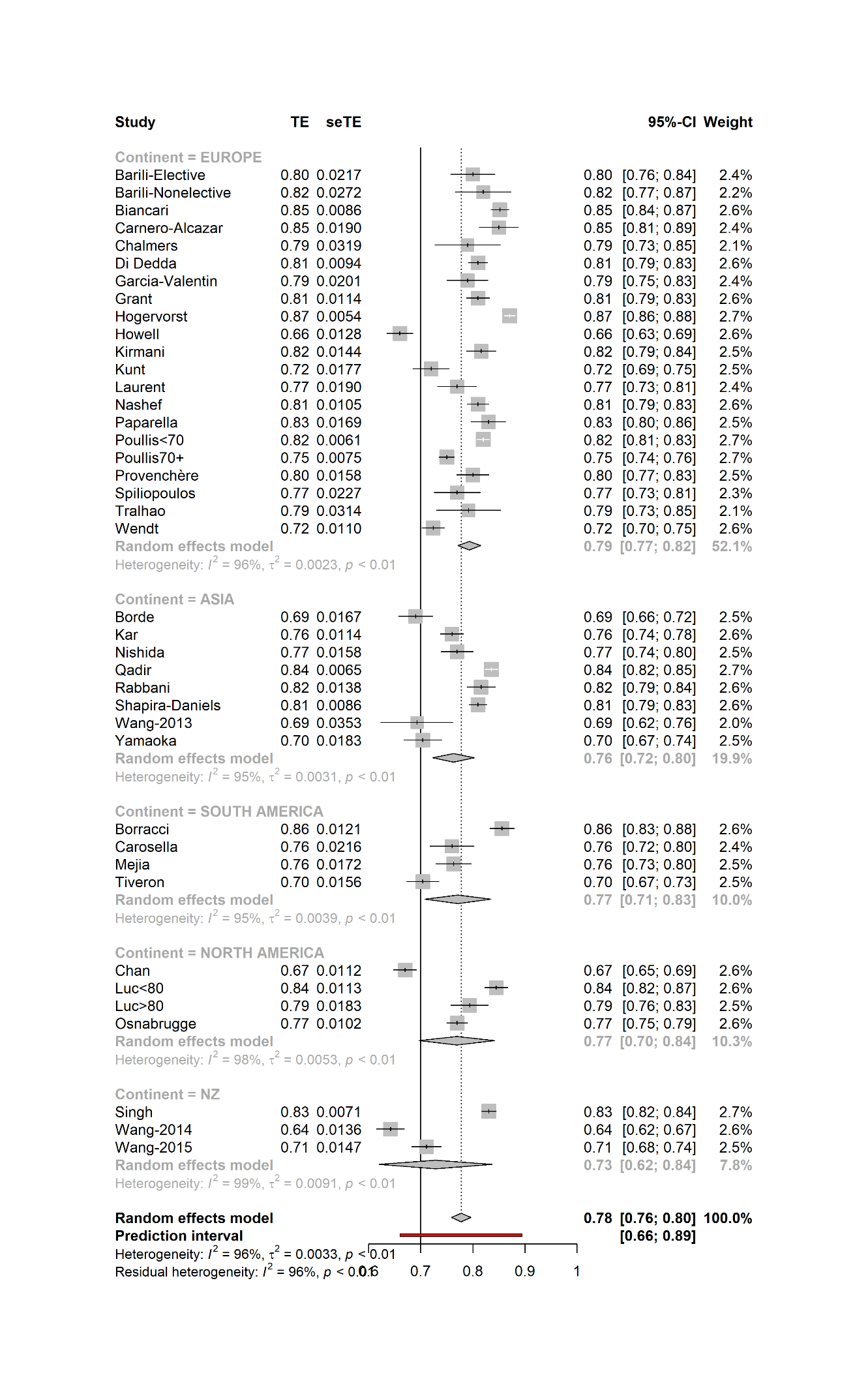


1. By presence of patients operated on before 2010


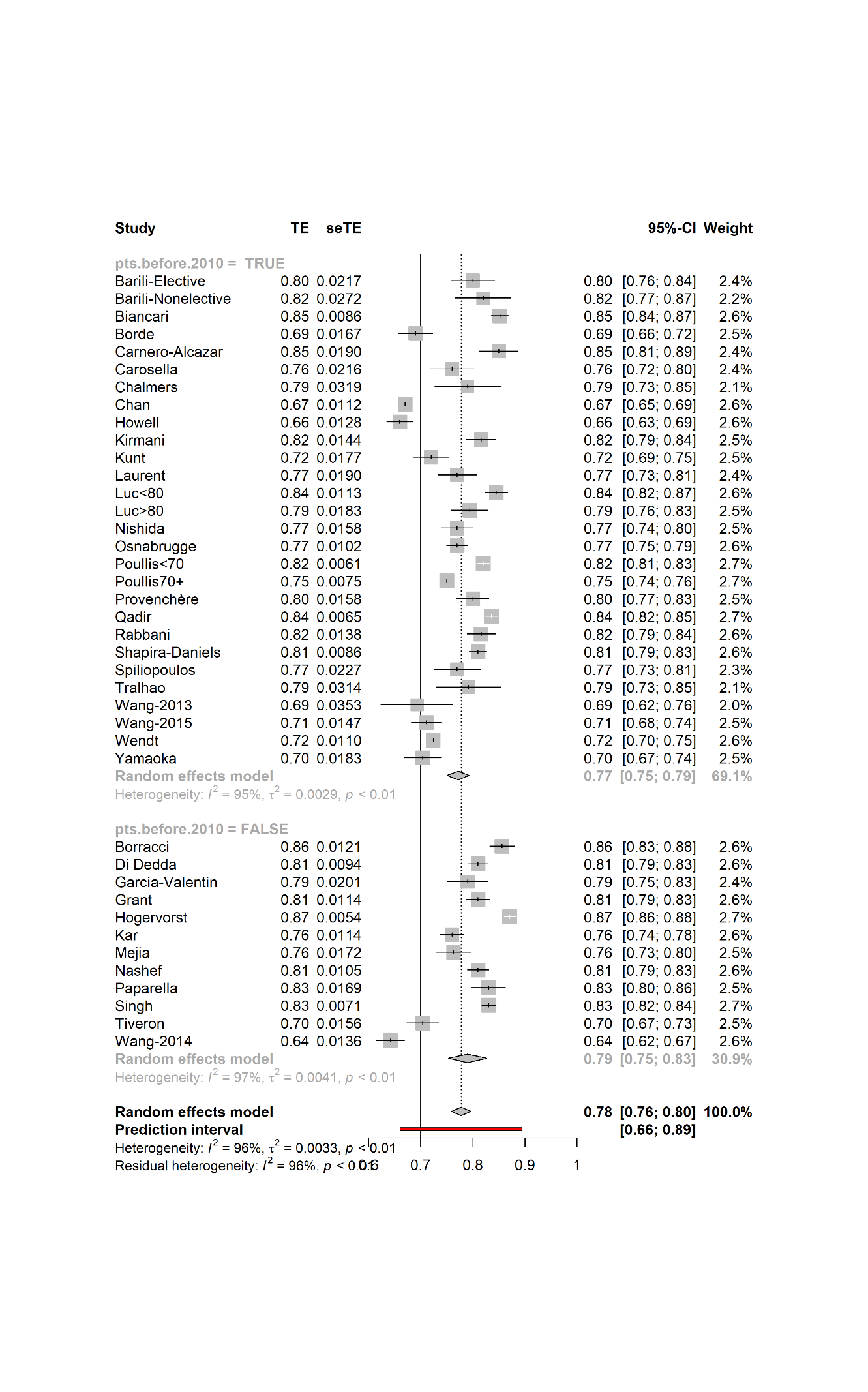


**Supplementary Figure 8. EuroSCORE2 –** Subgroup Analysis of Calibration. TE: O:E ratio. seTE: standard error of the O:E ratio

1. By Operation


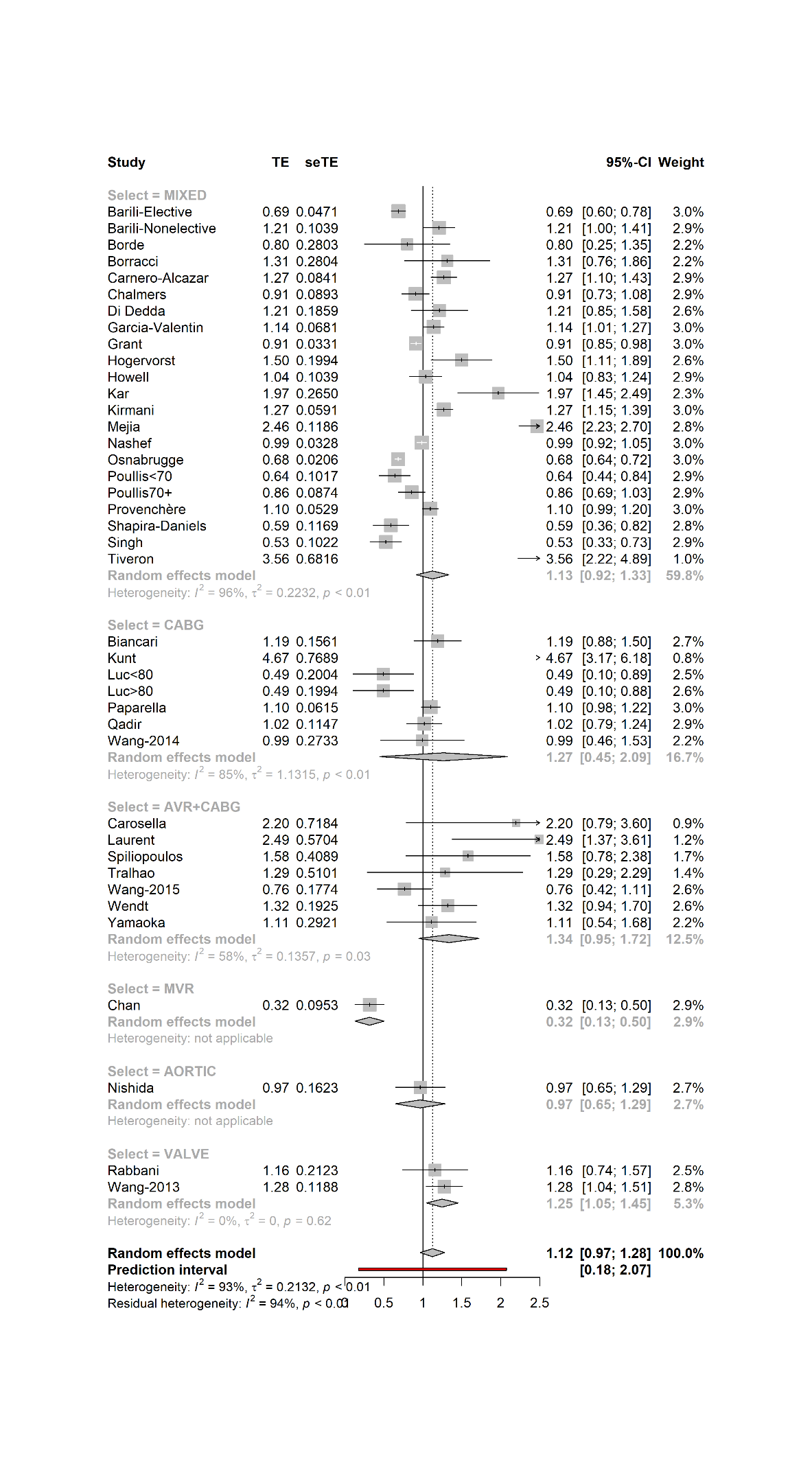


1. By Continent


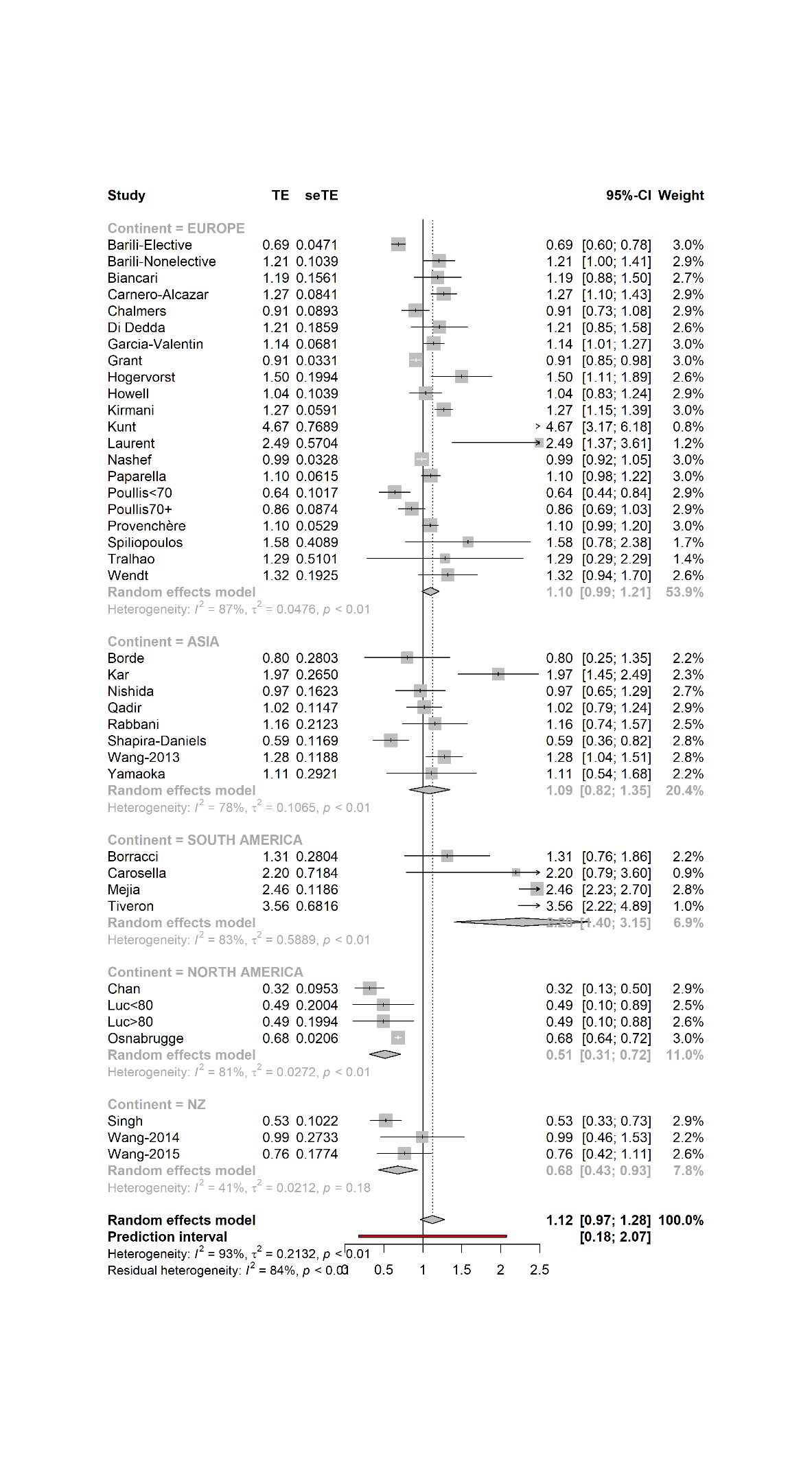


1. By presence of patients operated on before 2010


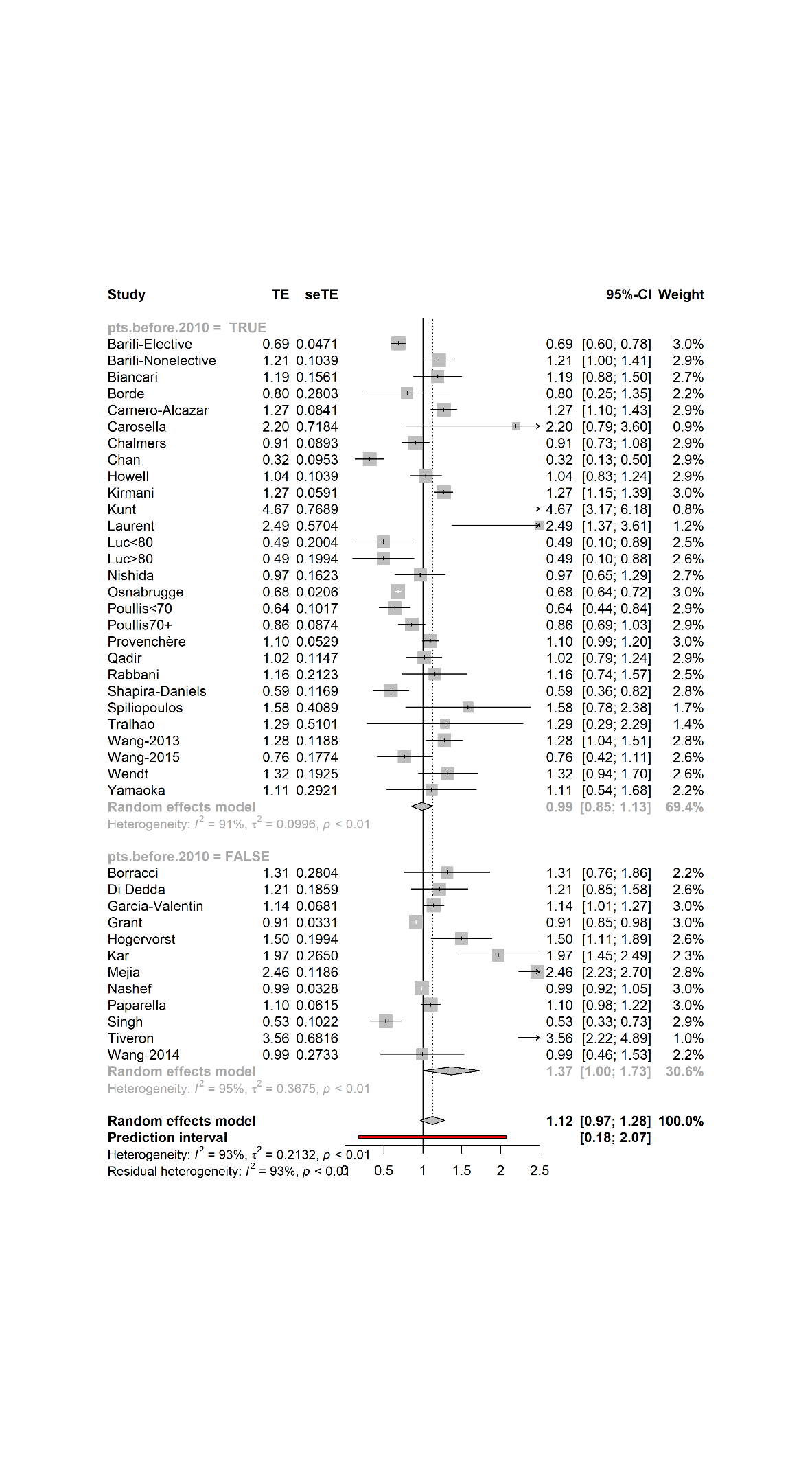


**Supplementary Figure 9. Society of Thoracic Surgeons Score –** Subgroup Analysis of Discrimination. AUC: c-statistic. SE: standard error of the c-statistic

1. By Operation


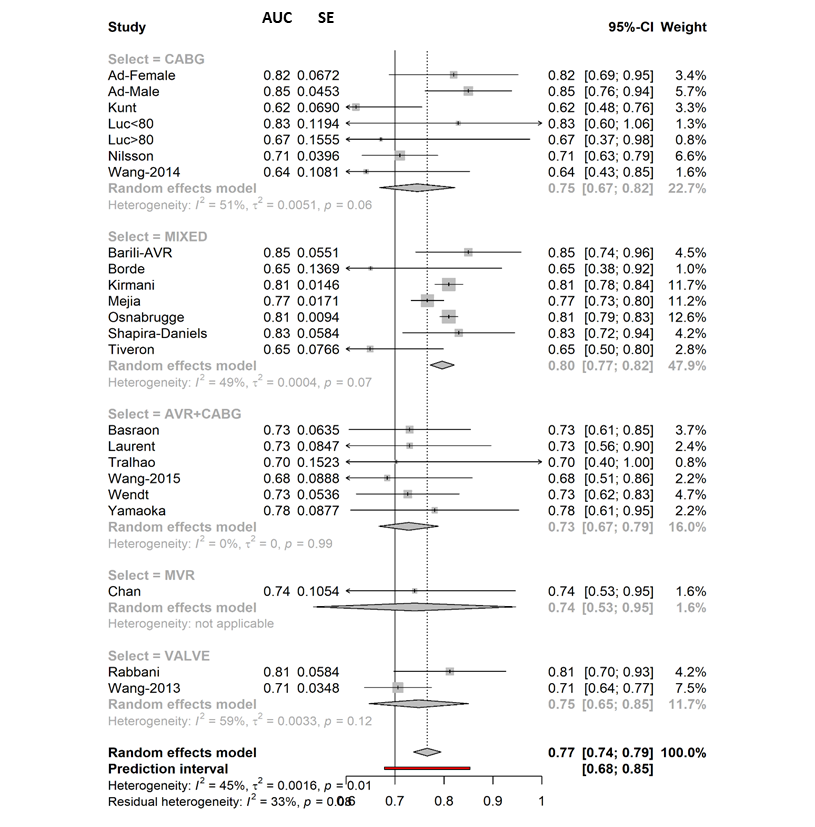


1. By Continent


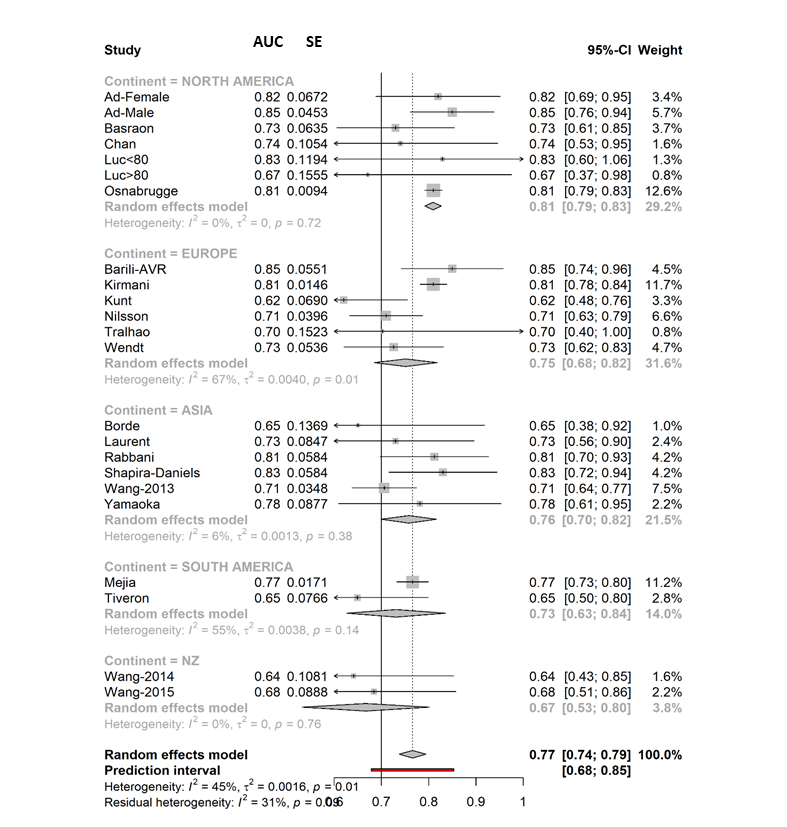


1. By presence of patients operated on before 2010

**
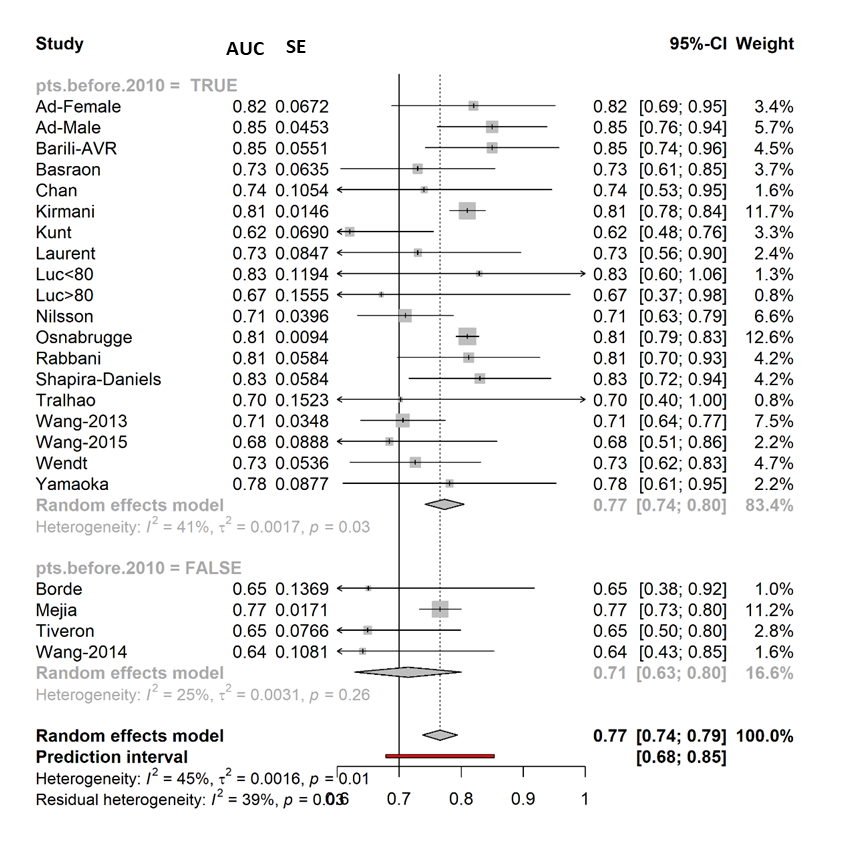
**

**Supplementary Figure 10. Society of Thoracic Surgeons Score –** Subgroup Analysis of Calibration. . TE: O:E ratio. seTE: standard error of the O:E ratio

By Operation


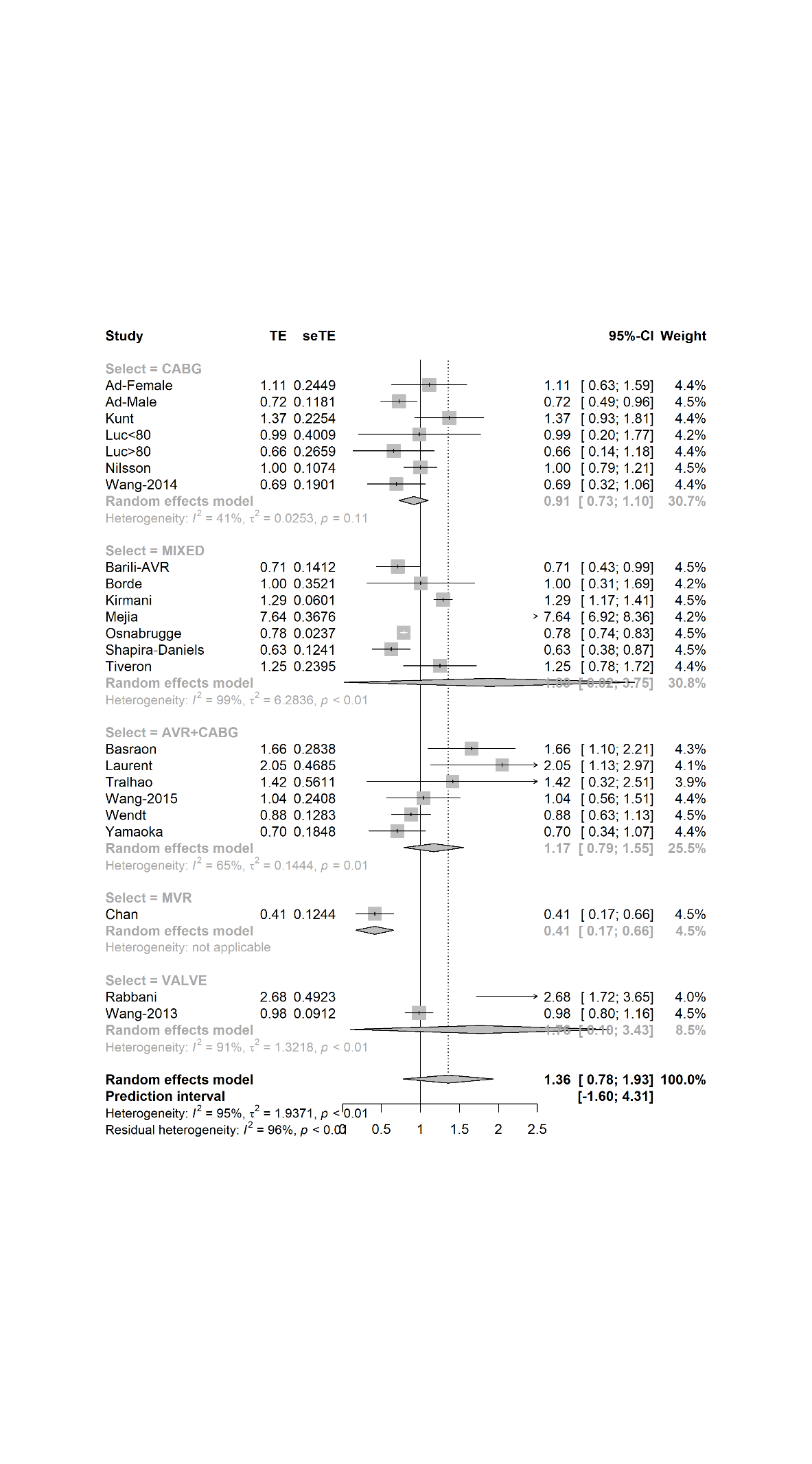


1. By Continent


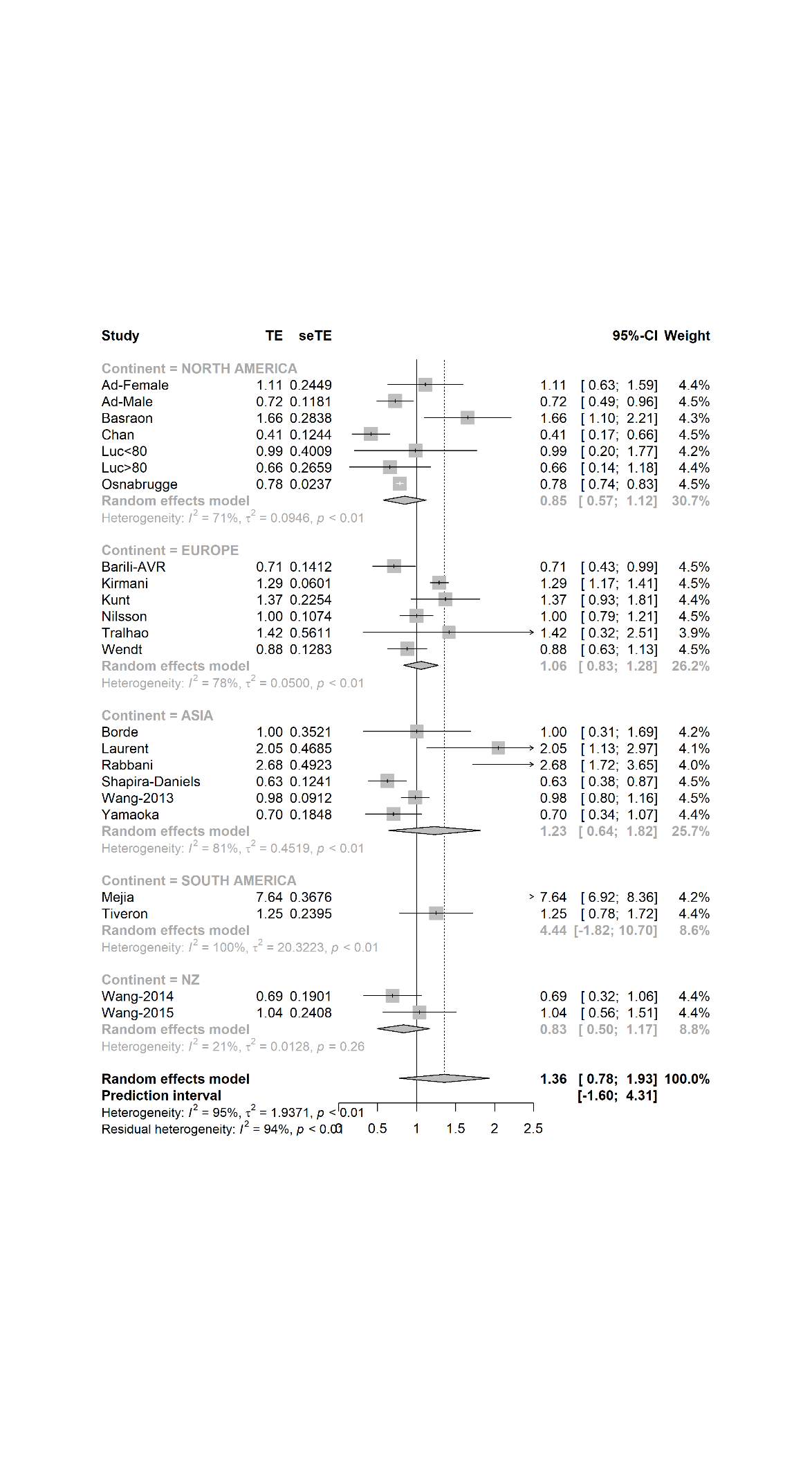


1. By presence of patients operated on before 2010


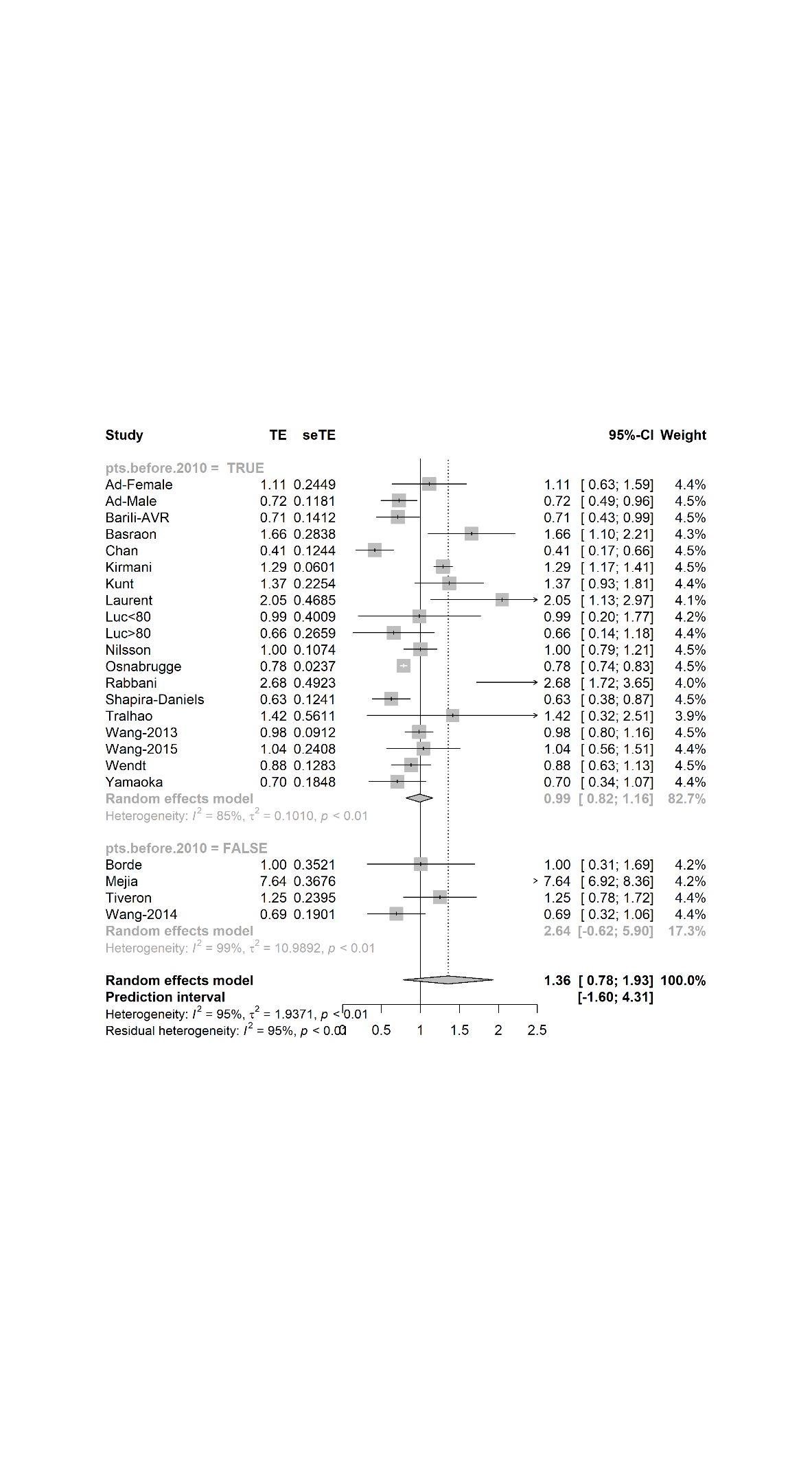


**References**

1. Prins C, Jonker IDV, Botes L, Smit FE. Cardiac surgery risk-stratification models. *Cardiovasc J Afr*. 2012;23:160–164.

2. Granton J, Cheng D. Risk stratification models for cardiac surgery. *Semin Cardiothorac Vasc Anesth*. 2008;12:167–174.

3. Stoica SC, Sharples LD, Ahmed I, Roques F, Large SR, Nashef SAM. Preoperative risk prediction and intraoperative events in cardiac surgery. *Eur J Cardio-thoracic Surg*. 2002;21:41–46.

4. Hickey GL, Grant SW, Caiado C, Kendall S, Dunning J, Poullis M, Buchan I, Bridgewater B. Dynamic prediction modeling approaches for cardiac surgery. *Circ Cardiovasc Qual Outcomes*. 2013;6:649–658.

5. Nashef SAM, Sharples LD, Roques F, Lockowandt U. EuroSCORE II and the art and science of risk modelling. *Eur J Cardio-thoracic Surg*. 2013;43:695–696.

6. Keogh BE. Logistic, additive or historical: Is EuroSCORE an appropriate model for comparing individual surgeons’ performance? *Heart*. 2006;92:1715–1716.

7. Hu Z, Chen S, Du J, Gu D, Wang Y, Hu S, Zheng Z. An In-hospital Mortality Risk Model for Patients Undergoing Coronary Artery Bypass Grafting in China. *Ann Thorac Surg*. 2020;109:1234–1242.

8. Barili F, Pacini D, Grossi C, Di Bartolomeo R, Alamanni F, Parolari A. Reliability of new scores in predicting perioperative mortality after mitral valve surgery. *J Thorac Cardiovasc Surg*. 2014;147:1008–1012.

9. Goetzenich A, Deppe I, Schnöring H, Gafencu GL, Gafencu DA, Yildirim H, Tewarie L, Spillner J, Moza A. EuroScore 2 for identification of patients for transapical aortic valve replacement - a single center retrospective in 206 patients. *J Cardiothorac Surg*. 2012;7:1–7.

10. Barili F, Di Gregorio O, Capo A, Ardemagni E, Rosato F, Argenziano M, Grossi C. Aortic valve replacement: Reliability of EuroSCORE in predicting early outcomes. *Int J Cardiol*. 2010;144:343–345.

11. Barmettler H, Immer FF, Berdat PA, Eckstein FS, Kipfer B, Carrel TP. Risk-stratification in thoracic aortic surgery: Should the EuroSCORE be modified? *Eur J Cardio-thoracic Surg*. 2004;25:691–694.

12. Shanmugam G, West M, Berg G. Additive and logistic EuroSCORE performance in high risk patients. *Interact Cardiovasc Thorac Surg*. 2005;4:299–303.

13. Kalavrouziotis D, Li D, Buth KJ, Légaré JF. The European System for Cardiac Operative Risk Evaluation (EuroSCORE) is not appropriate for withholding surgery in high-risk patients with aortic stenosis: A retrospective cohort study. *J Cardiothorac Surg*. 2009;4:1–8.

14. Ranucci M, Castelvecchio S, Menicanti L, Frigiola A, Pelissero G. Risk of assessing mortality risk in elective cardiac operations: Age, creatinine, ejection fraction, and the law of parsimony. *Circulation*. 2009;119:3053–3061.

15. Basraon J, Chandrashekhar YS, John R, Agnihotri A, Kelly R, Ward H, Adabag S. Comparison of risk scores to estimate perioperative mortality in aortic valve replacement surgery. *Ann Thorac Surg*. 2011;92:535–540.

16. Poullis M, Pullan M, Chalmers J, Mediratta N. The validity of the original EuroSCORE and EuroSCORE II in patients over the age of seventy. *Interact Cardiovasc Thorac Surg*. 2015;20:172–177.

17. Nashef SAM, Roques F, Sharples LD, Nilsson J, Smith C, Goldstone AR, Lockowandt U. Euroscore II. *Eur J Cardio-thoracic Surg*. 2012;41:734–745.

18. Grant SW, Hickey GL, Dimarakis I, Trivedi U, Bryan A, Treasure T, Cooper G, Pagano D, Buchan I, Bridgewater B. How does EuroSCORE II perform in UK cardiac surgery; an analysis of 23 740 patients from the Society for Cardiothoracic Surgery in Great Britain and Ireland National Database. *Heart*. 2012;98:1568–1572.

19. Chalmers J, Pullan M, Fabri B, McShane J, Shaw M, Mediratta N, Poullis M. Validation of EuroSCORE II in a modern cohort of patients undergoing cardiac surgery. *Eur J Cardio-thoracic Surg*. 2013;43:688–694.

20. Di Dedda U, Pelissero G, Agnelli B, De Vincentiis C, Castelvecchio S, Ranucci M. Accuracy, calibration and clinical performance of the new EuroSCORE II risk stratification system. *Eur J Cardiothorac Surg*. 2013;43:27–32.

21. Howell NJ, Head SJ, Freemantle N, van der Meulen TA, Senanayake E, Menon A, Kappetein AP, Pagano D. The new EuroSCORE II does not improve prediction of mortality in high-risk patients undergoing cardiac surgery: A collaborative analysis of two European centres. *Eur J Cardio-thoracic Surg*. 2013;44:1006–1011.

22. Biancari F, Vasques F, Mikkola R, Martin M, Lahtinen J, Heikkinen J. Validation of EuroSCORE II in patients undergoing coronary artery bypass surgery. *Ann Thorac Surg*. 2012;93:1930–1935.

23. Hogervorst EK, Rosseel PMJ, van de Watering LMG, Brand A, Bentala M, van der Meer BJM, van der Bom JG. Prospective validation of the EuroSCORE II risk model in a single Dutch cardiac surgery centre. *Netherlands Hear J*. 2018;26:540–551.

24. Provenchère S, Chevalier A, Ghodbane W, Bouleti C, Montravers P, Longrois D, Iung B. Is the EuroSCORE II reliable to estimate operative mortality among octogenarians? *PLoS One*. 2017;12:1–14.

25. Singh N, Gimpel D, Parkinson G, Conaglen P, Meikle F, Lin Z, Kejriwal N, Odom N, McCormack DJ, El-Gamel A. Assessment of the EuroSCORE II in a New Zealand Tertiary Centre. *Hear Lung Circ*. 2019;28:1670–1676.

26. Ad N, Barnett SD, Speir AM. The performance of the EuroSCORE and the Society of Thoracic Surgeons mortality risk score: The gender factor. *Interact Cardiovasc Thorac Surg*. 2007;6:192–195.

27. Barili F, Pacini D, Capo A, Ardemagni E, Pellicciari G, Zanobini M, Grossi C, Shahin KM, Alamanni F, Di Bartolomeo R, Parolari A. Reliability of new scores in predicting perioperative mortality after isolated aortic valve surgery: A comparison with the society of thoracic surgeons score and logistic EuroSCORE. *Ann Thorac Surg*. 2013;95:1539–1544.

28. Barili F, Pacini D, Rosato F, Roberto M, Battisti A, Grossi C, Alamanni F, Di Bartolomeo R, Parolari A. In-hospital mortality risk assessment in elective and non-elective cardiac surgery: A comparison between EuroSCORE II and age, creatinine, ejection fraction score. *Eur J Cardio-thoracic Surg*. 2014;46:44–48.

29. Carnero-Alcázar M, Guisasola JAS, Lacruz FJR, Castellanos LCM, Carnicer JC, Medinilla EV, Sánchez TT, Hernández JER. Validation of EuroSCORE II on a single-centre 3800 patient cohort. *Interact Cardiovasc Thorac Surg*. 2013;16:293–300.

30. Borracci RA, Rubio M, Celano L, Ingino CA, Allende NG, Guerrero RAA. Prospective validation of EuroSCORE II in patients undergoing cardiac surgery in Argentinean centres. *Interact Cardiovasc Thorac Surg*. 2014;18:539–543.

31. Carosella V, Mastantuono C, Golovonevsky V, Cohen V, Grancelli H, Rodriguez W, Cardenas C, Nojek C. Prospective and Multicentric Validation of the ArgenSCORE in Aortic Valve Replacement Surgery. Comparison with the EuroSCORE I and the EuroSCORE II. *Rev Argent Cardiol*. 2014;82. doi:10.7775/rac.v82.i2.3388.

32. Chan V, Ahrari A, Ruel M, Elmistekawy E, Hynes M, Mesana TG. Perioperative deaths after mitral valve operations may be overestimated by contemporary risk models. *Ann Thorac Surg*. 2014;98:605–610.

33. Nishida T, Sonoda H, Oishi Y, Tanoue Y, Nakashima A, Shiokawa Y, Tominaga R. The novel EuroSCORE II algorithm predicts the hospital mortality of thoracic aortic surgery in 461 consecutive Japanese patients better than both the original additive and logistic EuroSCORE algorithms. *Interact Cardiovasc Thorac Surg*. 2014;18:446–450.

34. Paparella D, Guida P, Di Eusanio G, Caparrotti S, Gregorini R, Cassese M, Fanelli V, Speziale G, Mazzei V, Zaccaria S, De Luca Tupputi Schinosa L, Fiore T. Risk stratification for in-hospital mortality after cardiac surgery: External validation of EuroSCORE II in a prospective regional registry. *Eur J Cardio-thoracic Surg*. 2014;46:840–848.

35. Spiliopoulos K, Bagiatis V, Deutsch O, Kemkes BM, Antonopoulos N, Karangelis D, Haschemi A, Gansera B. Performance of EuroSCORE II compared to EuroSCORE I in predicting operative and mid-term mortality of patients from a single center after combined coronary artery bypass grafting and aortic valve replacement. *Gen Thorac Cardiovasc Surg*. 2014;62:103–111.

36. Garcia-Valentin A, Mestres CA, Bernabeu E, Bahamonde JA, Martín I, Rueda C, Domenech A, Valencia J, Fletcher D, Machado F, Amores J. Validation and quality measurements for EuroSCORE and EuroSCORE II in the Spanish cardiac surgical population: A prospective, multicentre study. *Eur J Cardio-thoracic Surg*. 2016;49:399–405.

37. Kar P, Geeta K, Gopinath R DP. Mortality prediction in Indian cardiac surgery patients: Validation of European System for Cardiac Operative Risk Evaluation II. *Indian J Anaesth*. 2017;61:157–162.

38. Kirmani BH, Mazhar K, Fabri BM, Pullan DM. Comparison of the EuroSCORE II and Society of Thoracic Surgeons 2008 risk tools. *Eur J Cardio-thoracic Surg*. 2013;44:999–1005.

39. Borde D, Gandhe U, Hargave N, Pandey K K V. The application of European system for cardiac operative risk evaluation II (EuroSCORE II) and Society of Thoracic Surgeons (STS) risk-score for risk stratification in Indian patients undergoing cardiac surgery. *Ann Card Anaesth*. 2013;16:163–166.

40. Kunt AG, Kurtcephe M, Hidiroglu M, Cetin L, Kucuker A, Bakuy V, Ruchan Akar A, Sener E. Comparison of original EuroSCORE, EuroSCORE II and STS risk models in a Turkish cardiac surgical cohort. *Interact Cardiovasc Thorac Surg*. 2013;16:625–629.

41. Laurent M, Fournet M, Feit B, Oger E, Donal E, Thébault C, Biron Y, Beneux X, Sellin M, Le Reveillé S, Flecher E, Leguerrier A. Simple bedside clinical evaluation versus established scores in the estimation of operative risk in valve replacement for severe aortic stenosis. *Arch Cardiovasc Dis*. 2013;106:651–660.

42. Luc JGY, Graham MM, Norris CM, Al Shouli S, Nijjar YS, Meyer SR. Predicting operative mortality in octogenarians for isolated coronary artery bypass grafting surgery: A retrospective study. *BMC Cardiovasc Disord*. 2017;17:1–7.

43. Vilca Mejia OA, Borgomoni GB, Zubelli JP, Palma Dallan LR, Alberto Pomerantzeff PM, Praça Oliveira MA, Junior OP, Gradim Tiveron M, Arruda Nakazone M, Tineli RÂ, Campagnucci VP, Rocha e Silva R, Rodrigues AJ, Gomes WJ, Ferreira Lisboa LA, Jatene FB. Validation and quality measurements for STS, EuroSCORE II and a regional risk model in Brazilian patients. *PLoS One*. 2020;15:1–16.

44. Nilsson J, Algotsson L, Höglund P, Lührs C, Brandt J. Early mortality in coronary bypass surgery: The EuroSCORE versus the Society of Thoracic Surgeons risk algorithm. *Ann Thorac Surg*. 2004;77:1235–1239.

45. Osnabrugge RL, Speir AM, Head SJ, Fonner CE, Fonner E, Kappetein AP, Rich JB. Performance of EuroSCORE II in a large US database: Implications for transcatheter aortic valve implantation. *Eur J Cardio-thoracic Surg*. 2014;46:400–408.

46. Qadir I, Alamzaib SM, Ahmad M, Perveen S, Sharif H. EuroSCORE vs. EuroSCORE II vs. society of thoracic surgeons risk algorithm. *Asian Cardiovasc Thorac Ann*. 2014;22:165–171.

47. Rabbani MS, Qadir I, Ahmed Y, Gul M, Sharif H. Heart valve surgery: EuroSCORE vs. EuroSCORE II vs. Society of thoracic surgeons score. *Heart Int*. 2014;9:53–58.

48. Shapira-Daniels A, Blumenfeld O, Korach A, Rudis E, Izhar U, Shapira OM. The American society of thoracic surgery score versus euroscore i and euroscore II in israeli patients undergoing cardiac surgery. *Isr Med Assoc J*. 2019;21:671–675.

49. Tiveron MG, Bomfim HA, Simplício MS, Bergonso MH, De Matos MPB, Ferreira SM, Pelloso EA, De Barros RT. Desempenho do InsCor e de três escores internacionais em cirurgia cardíaca na Santa Casa de Marília. *Brazilian J Cardiovasc Surg*. 2015;30:1–8.

50. Tralhão A, Campante Teles R, Sousa Almeida M, Madeira S, Borges Santos M, Andrade MJ, Mendes M, Neves JP. Aortic valve replacement for severe aortic stenosis in octogenarians: Patient outcomes and comparison of operative risk scores. *Rev Port Cardiol*. 2015;34:439–446.

51. Wang C, Li X, Lu F lin, Xu J bin, Tang H, Han L, Xu Z yun. Comparison of six risk scores for in-hospital mortality in Chinese patients undergoing heart valve surgery. *Hear Lung Circ*. 2013;22:612–617.s

52. Wang TKM, Li AY, Ramanathan T, Stewart RAH, Gamble G, White HD. Comparison of four risk scores for contemporary isolated coronary artery bypass grafting. *Hear Lung Circ*. 2014;23:469–474.

53. Wang TKM, Choi DHM, Stewart R, Gamble G, Haydock D, Ruygrok P. Comparison of four contemporary risk models at predicting mortality after aortic valve replacement. *J Thorac Cardiovasc Surg*. 2015;149:443–448.

54. Wendt D, Thielmann M, Kahlert P, Kastner S, Price V, Al-Rashid F, Patsalis P, Erbel R, Jakob H. Comparison between different risk scoring algorithms on isolated conventional or transcatheter aortic valve replacement. *Ann Thorac Surg*. 2014;97:796–802.

55. Yamaoka H, Kuwaki K, Inaba H, Yamamoto T, Kato TS, Dohi S, Matsushita S, Amano A. Comparison of modern risk scores in predicting operative mortality for patients undergoing aortic valve replacement for aortic stenosis. *J Cardiol*. 2016;68:135–140.
